# Supplementary material for: Template-assisted sequence knockin rescues skeletal and cardiac muscle function in a deletion model of Duchenne muscular dystrophy
Source: Mol Ther. 2025 May 7;33(8):3733–45. doi: 10.1016/j.ymthe.2025.05.005 (PMC12461635; doi:10.1016/j.ymthe.2025.05.005)
Supplement: Supplementary file 1 [file mmc1.pdf]

**YMTHE, Volume *33***

## Supplemental Information

# Template-assisted sequence knockin rescues skeletal and cardiac muscle function in a deletion model of Duchenne muscular dystrophy

**Sina Fatehi, Matthew J. Rok, Ryan M. Marks, Emily Huynh, Natalie Kozman, Hong Anh Truong, Lijun Chi, Bei Yan, Enzhe Khazeeva, Paul Delgado-Olguin, Evgueni A. Ivakine, and Ronald D. Cohn**

## Table S1. sgRNA sequence for the TASK system

| sgRNA name | Spacer sequence (5'-3') | PAM sequence (5'-3') |
|---|---|---|
| TASK-in54g3 | CATGATCTTGGCATATGGAG | TGGAGT |

**Table S2. TASK-in54g3 top off target sites and PCR/sequencing primers.** Forward primer was used in the Sanger sequencing reaction.

| Sequence | Sequence (5'-3') | PAM sequence (5'-3') |
|---|---|---|
| OT1 protospacer sequence | CATGAGCTATGCATATGGAG | ATGAAT |
| **Forward primer** | **TGCTGAGTGTAAGCAGCTATC** | |
| Reverse primer | TTCAAGCACAGAGCACACTC | |
| OT2 protospacer sequence | CATGTTCTGGGCATCTGGAG | GGGAGT |
| **Forward primer** | **ACCTGAGATGCAGTCTGTTGAG** | |
| Reverse primer | TCAAGTCCTTGGGACTGTTGG | |
| OT3 protospacer sequence | CAAGATCCTGGCATATGGGG | GTGGGT |
| **Forward primer** | **GTCTTCCCTCATCCACACCC** | |
| Reverse primer | ACCTCCATACACACATGCTGG | |

## Table S3. Genomic and cDNA PCR primer designs

| Purpose | Primer name | Primer Sequence (5’-3’) |
|---|---|---|
| Amplify the TASK-mediated *Dmd* exon 52-54 SSA integration product from treated C2C12 cells and muscle tissue following intramuscular injections in *Dmd Δ52-54* mice. | Dmd_d52-54_gKI_F | GAG ATT GGG TTC ACA GAA GGC |
| | Dmd_d52-54_gKI_R | TGT CCT ATG ACC TGT TCG GC |
| Amplify the edited transcript, containing an extra copy of exons 52-54 in TASK treated C2C12 cells. | Dmd_Ex52-54_int_F2 | CTTGGGGAAACATTCATAAAAGGC |
| | Dmd_Ex52-54_int_R2 | TTGTAACAGGGGTGCTTCATCC |
| Amplify the wildtype *Dmd* transcript, following intramuscular injections in *Dmd Δ52-54* mice. | mus_Dmd_ex51-F2 | TGG GTG ATC TGG AAG ACA TCA ATG |
| | m_DMD_qPCR_ex52-R | GTT CTA GCT TCT TGA TTG CTG GTT TTG |
| Amplify the TASK-mediated *Dmd* exon 52-54 SSA and NHEJ integration products from muscle tissue following systemic injections in *Dmd Δ52-54* mice. | HMEJ-d52-54-KI Fw #2 | ATTCCGTGTCTGTCTGTGCAT |
| | HMEJ-d52-54-KI RV #2 | GGGCGTCTCTGTTCCAAATC |

## Table S4. dPCR assay primer and probe designs

| Purpose | Primer/probe name | Primer/probe sequence (5’-3’) |
|---|---|---|
| Assay to quantify wildtype *Dmd* transcripts. | CDXGRUV_F | CACAGAAACCAAGCAGTTGGC |
| | CDXGRUV_R | TCTTCCAAAGCAGCCTCTTGC |
| | CDXGRUV_P (probe) | ACGTGGCAAATGAT |
| Assay to quantify TASK integration events from genomic DNA. | HMEJ_del52-54_KI_ddPCR_F | GCTAAGACAGCATTACATTGA |
| | HMEJ_del52-54_KI_ddPCR_R | TTAAACCCGGCAAAAGCATA |
| | HMEJ_del52-54_Ki_ddPCR_Probe (probe) | CACAGATAATAGAGGACTTC |
| Reference primer/probe for both the assays listed above | ddpcr_m_ex51_F | TGCCATCTTCTTTGCTGTTG |
| | ddpcr_m_ex51_R | GTCCAAGCTCGGTTGAAGTC |
| | APGZHP9_V VIC (probe) | TACCTGCACTGGCAGAC |

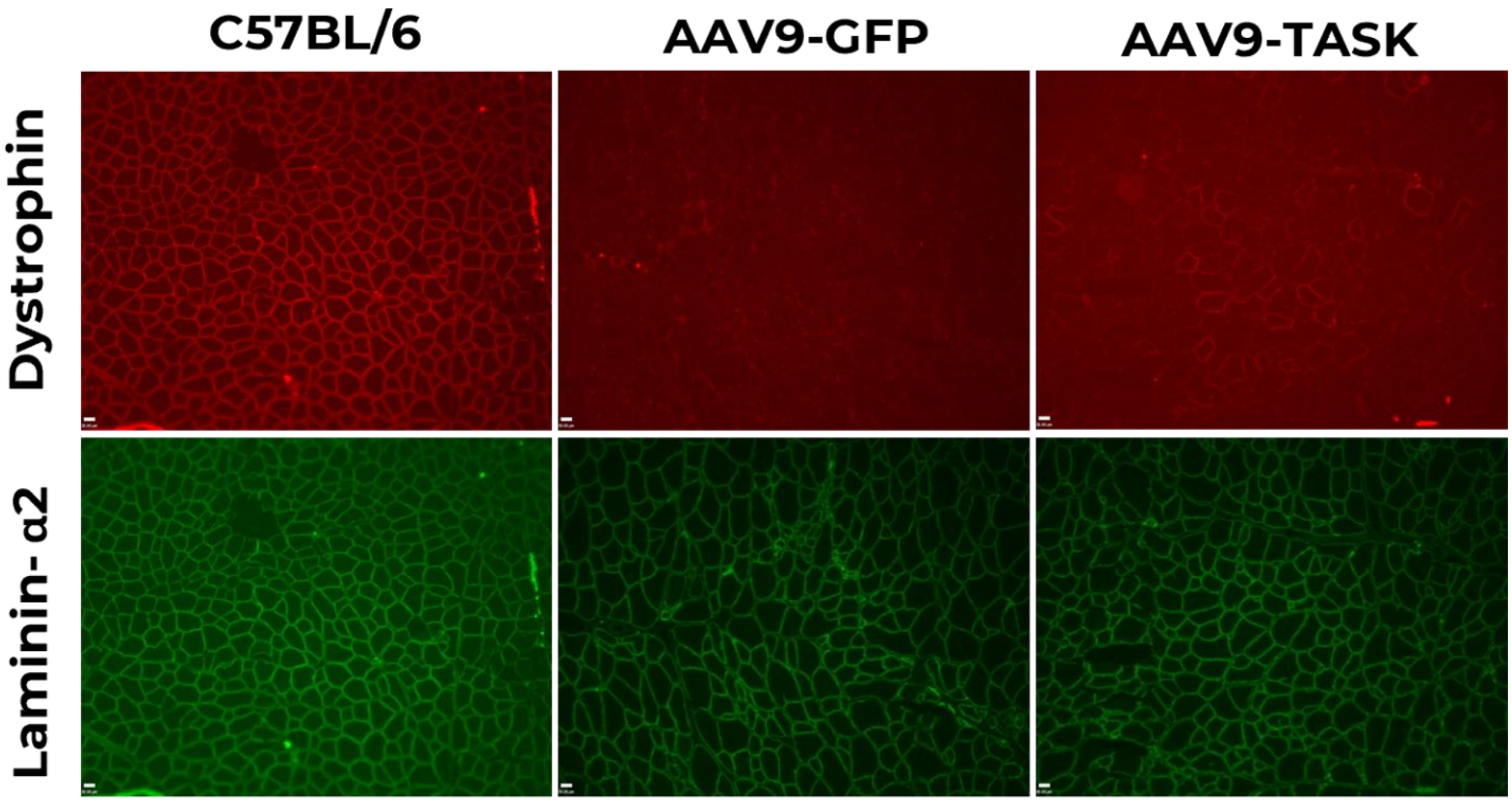


**Figure S1: Dystrophin and laminin-α2 immunofluorescence staining shows the presence of dystrophin-positive fibers in the tibialis anterior of treated mice (scale bar, 30 µm).**

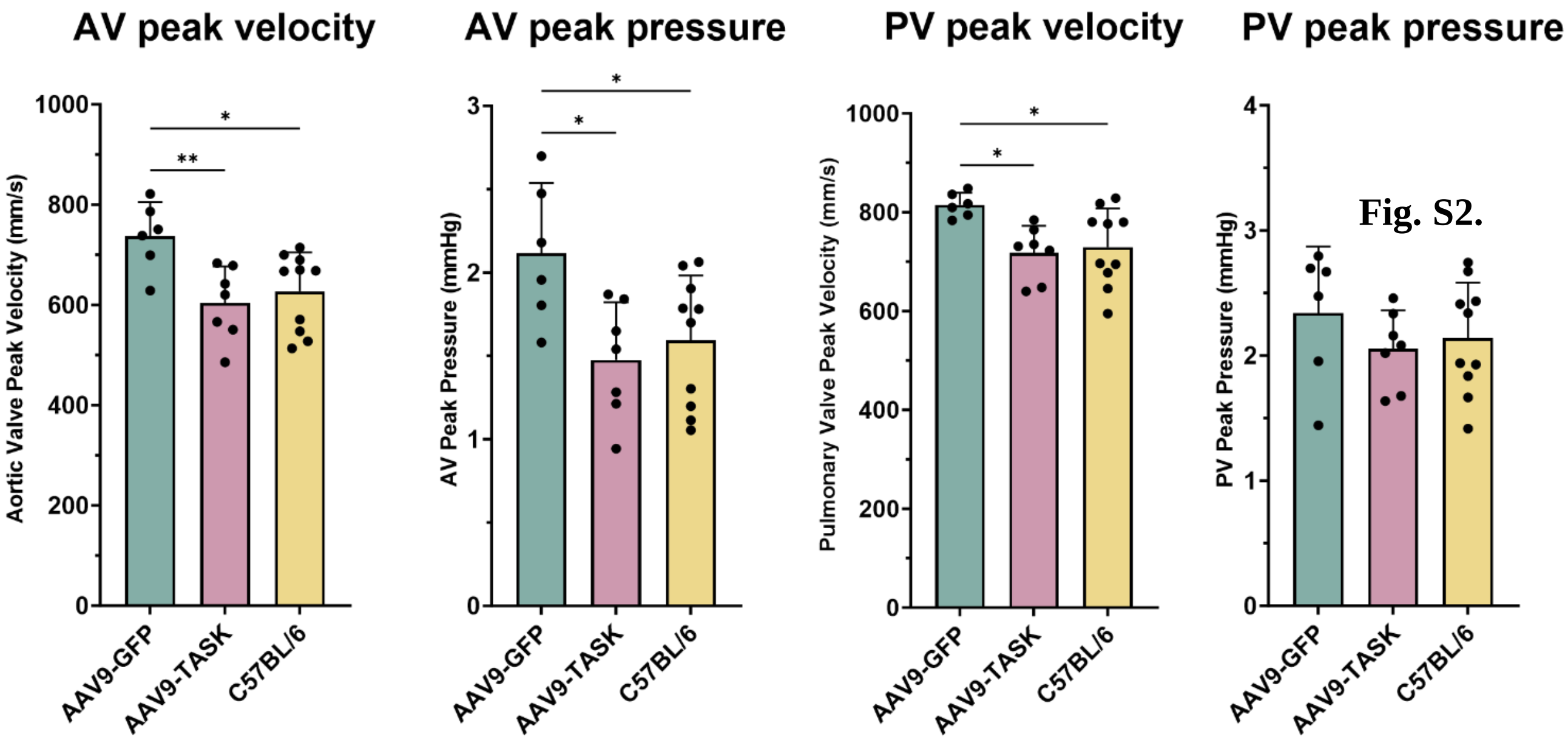


**Figure S2: Additional measurements of heart function indicating improvements in AAV9-TASK-treated animals.** One-way ANOVA, followed by Dunnett's post hoc multiple Comparisons test (∗∗∗∗p < 0.0001, ∗∗∗p < 0.001, ∗∗p < 0.01 and ∗p < 0.05; mean ± SD; n = 6-10 mice).

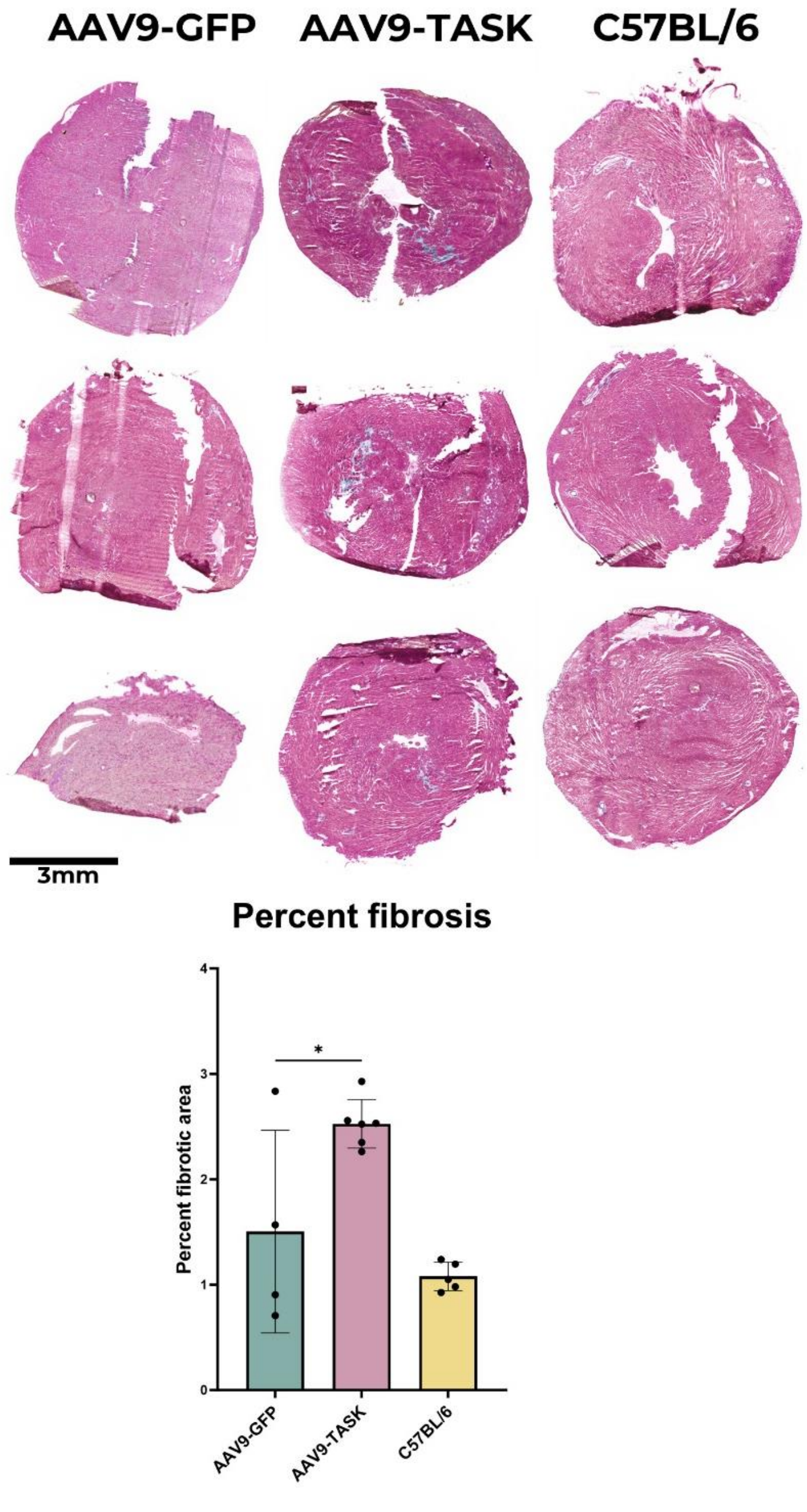


**Figure S3: Heart cryosections stained with Masson's trichrome and total fibrotic area quantified (scale bar, 3mm, each dot represents mean of 3-5 images per mouse).** One-way ANOVA, followed by Dunnett's post hoc multiple comparisons test (∗∗∗∗$p < 0.0001$, ∗∗∗$p < 0.001$, ∗∗$p < 0.01$ and ∗$p < 0.05$; mean ± SD; n = 4-6 mice).

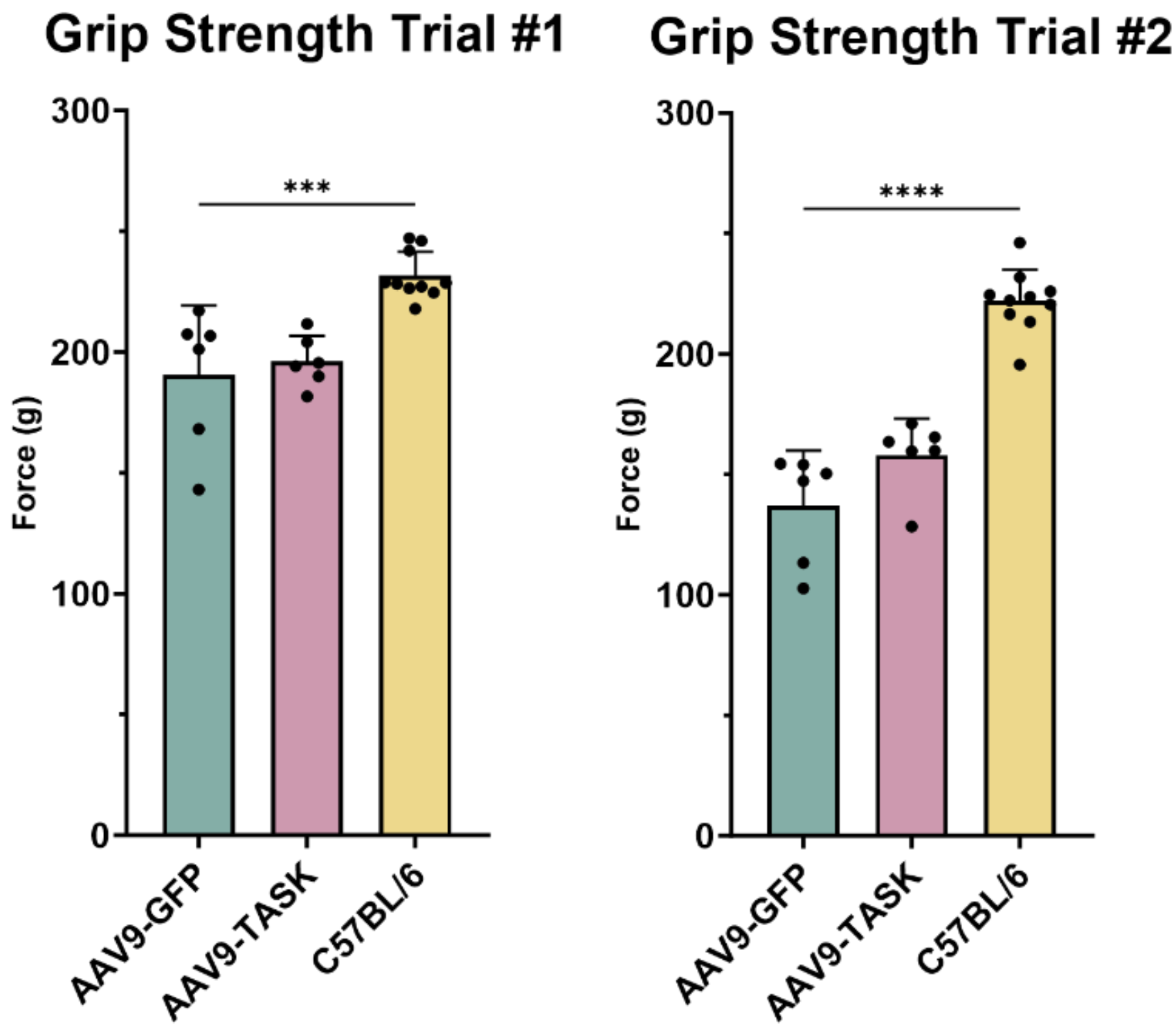


**Figure S4: Non-normalized forelimb and hindlimb grip strength test of AAV9-TASK-treated animals.** One-way ANOVA, followed by Dunnett's post hoc multiple comparisons test (∗∗∗∗$p < 0.0001$, ∗∗∗$p < 0.001$, ∗∗$p < 0.01$ and ∗$p < 0.05$; mean ± SD; n = 6-10 mice).

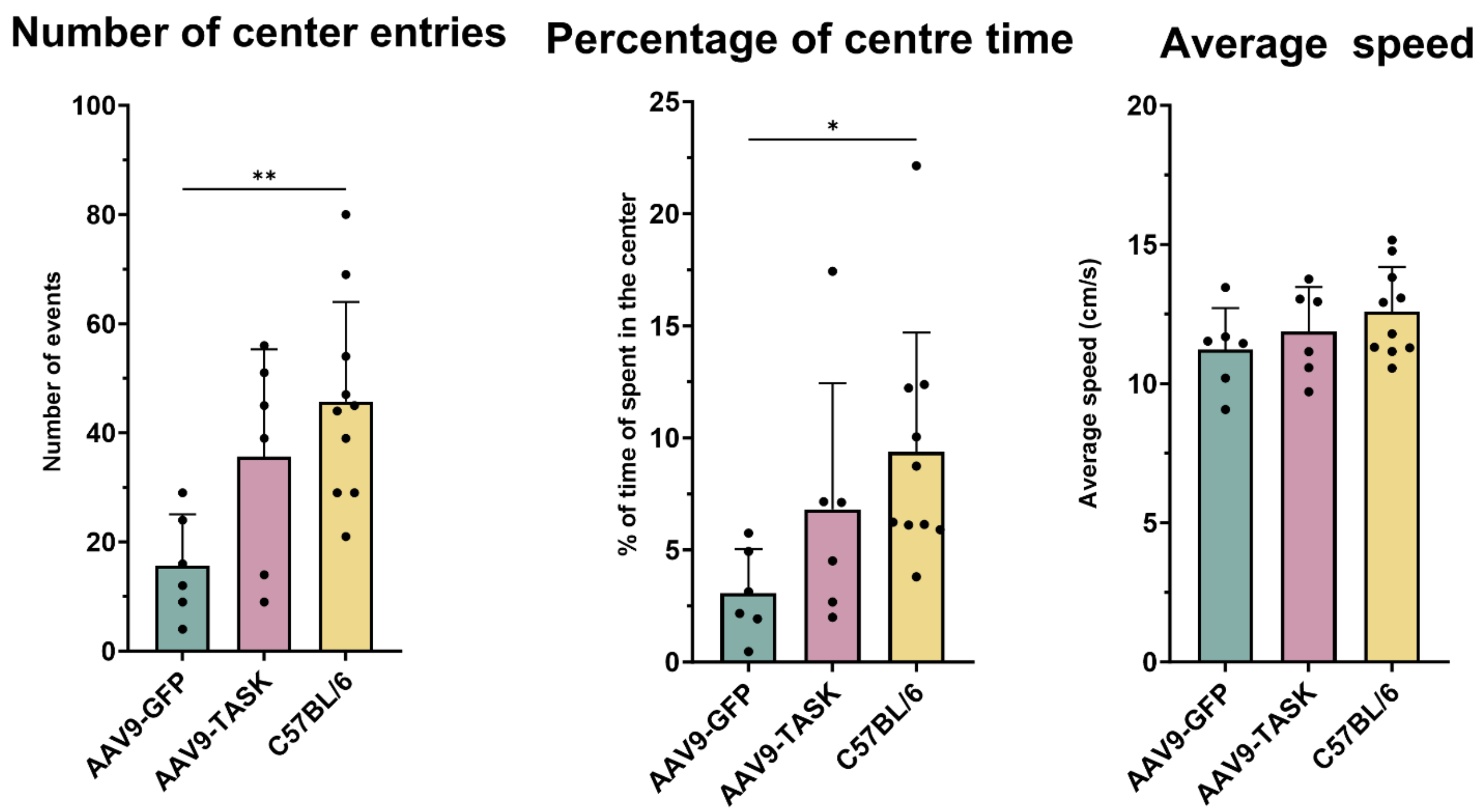


**Figure S5: Additional open field test results indicate positive trends in mobility for systemically treated *Dmd Δ52-54* mice.** One-way ANOVA, followed by Dunnett's post hoc multiple comparisons test (∗∗∗∗p < 0.0001, ∗∗∗p < 0.001, ∗∗p < 0.01 and ∗p < 0.05; mean ± SD; n = 6-10 mice).

**Movie S1.**
**B mode video of C57BL/6 mouse heart (left ventricle).**

**Movie S2.**
**B mode video of AAV9-GFP treated *Dmd Δ52-54* mouse (left ventricle).**

**Movie S3.**
**B mode video of AAV9-TASK treated *Dmd Δ52-54* mouse (left ventricle).**
