# Supplementary material for: Template-assisted sequence knockin rescues skeletal and cardiac muscle function in a deletion model of Duchenne muscular dystrophy
Source: Mol Ther. 2025 May 7;33(8):3733–45. doi: 10.1016/j.ymthe.2025.05.005 (PMC12461635; doi:10.1016/j.ymthe.2025.05.005)
Supplement: Supplementary file 2 [file mmc5.pdf]

Molecular Therapy
Original Article

Template-assisted sequence knockin rescues skeletal and cardiac muscle function in a deletion model of Duchenne muscular dystrophy

Sina Fatehi,[1,3] Matthew J. Rok,[1,3] Ryan M. Marks,[1,3] Emily Huynh,[1] Natalie Kozman,[1] Hong Anh Truong,[1] Lijun Chi,[2] Bei Yan,[2] Enzhe Khazeeva,[1,3] Paul Delgado-Olguin,[2,3] Evgueni A. Ivakine,[1,3,4] and Ronald D. Cohn[1,3,5]

[1]Program in Genetics and Genome Biology, Peter Gilgan Centre for Research and Learning, Toronto, ON M5G 0A4, Canada; [2]Program in Translational Medicine, Peter Gilgan Centre for Research and Learning, Toronto, ON M5G 0A4, Canada; [3]Department of Molecular Genetics, University of Toronto, Toronto, ON M5S 1A1, Canada; [4]Department of Physiology, University of Toronto, Toronto, ON M5S 1A1, Canada; [5]Department of Pediatrics, The Hospital for Sick Children, Toronto, ON M5G 1E8, Canada

Duchenne muscular dystrophy (DMD) poses challenges in therapy design due to dystrophin's complex role in maintaining muscle function since the restoration of truncated protein products has failed to completely address the disease's pathophysiology in clinical trials. As ∼70% of patients harbor deletions, strategies enabling targeted DNA insertion to restore full-length dystrophin protein are essential. Here, we present template-assisted sequence knockin (TASK), a strategy that we employed to specifically correct the *Dmd Δ52-54* mutation in a murine model. By co-delivering a repair template and the Cas9 nuclease using AAV9s, the splice-competent sequence for *Dmd* exons 52–54 was integrated into the residual intron 54 locus, resulting in the systemic restoration of full-length dystrophin at therapeutically relevant levels in the heart and across all skeletal muscles, leading to significant functional improvements. TASK demonstrates the highest efficiency of exogenous DNA knockin reported to date, achieving rescue of key dystrophic hallmarks in a deletion model of DMD.

INTRODUCTION

Advancements in treating the underlying causes of genetic disorders are offered by genome editing technologies. The use of CRISPR-Cas9 tools has significantly enhanced our ability to manipulate the genome *in vivo*; however, it remains limited in its capacity to perform large, targeted insertions, especially in non-dividing cells. The correction of large deletions frequently observed in patients with Duchenne muscular dystrophy (DMD) is one area where these treatments could provide substantial benefits. Affecting 1 in 5,000–1 in 6,000 live male newborns, DMD is the most common lethal genetically inherited pediatric disease having a significant impact on life expectancy and quality.[1–3] DMD is characterized by the absence of dystrophin protein in muscle cells, crucial for the formation of the dystrophin-associated protein complex (DAPC), which links the actin cytoskeleton of muscle cells to the extracellular matrix. The DAPC stabilizes cells and protects them from damage during cycles of contraction and relaxation. Loss of dystrophin results in sarcolemma fragility, leading to the accumulation of damage during cycles of contraction and relaxation. Currently proposed therapeutic strategies for DMD, such as exon skipping and micro-dystrophin gene therapy, which result in the production of truncated dystrophin products, have generated suboptimal results in the clinic.[4–10] While these truncated proteins do provide some benefits, they often lack key domains necessary to realize the full protective effects of dystrophin, leading to limited benefits in muscle function and health. This issue is particularly important when designing strategies to correct large, multi-exonic deletions, which account for approximately 70% of DMD cases, and where complete restoration would require reintegration of the missing coding sequences.[11,12]

Deletions in DMD are a crucial target for therapeutic intervention, and several approaches have been developed to address them. One group of strategies, known as exon skipping, removes out-of-frame exons from the mRNA transcript to restore the *DMD* gene's open reading frame and expression. Antisense oligonucleotides, exon excision, and CRISPR-Cas9-mediated splice site disruption using nucleases and base have been employed to accomplish this.[13–16] Another strategy called exon-reframing uses a Cas9 nuclease to induce double-strand breaks (DSBs) in an out-of-frame exon, which results in random insertions or deletions produced by the non-homologous end joining (NHEJ) repair pathway to realign the reading frame.[17] Despite this, a critical limitation remains across these approaches in that they do not restore the wild-type dystrophin protein. Expression of the complete dystrophin protein is necessary to fully address the functional deficits and clinical symptoms associated with DMD.

Received 3 March 2025; accepted 2 May 2025;
https://doi.org/10.1016/j.ymthe.2025.05.005.

Correspondence: Evgueni A. Ivakine, Program in Genetics and Genome Biology, Peter Gilgan Centre for Research and Learning, Toronto, ON M5G 0A4, Canada.
E-mail: zhenya.ivakine@sickkids.ca

© 2025 The Authors.
Published by Elsevier Inc. on behalf of The American Society of Gene and Cell Therapy.
This is an open access article under the CC BY license (http://creativecommons.org/licenses/by/4.0/).

An alternative to these approaches is to correct multi-exonic deletions like those in the *DMD* gene by performing exogenous DNA insertions. Non-dividing cells rely on two pathways to repair DSBs: NHEJ and single-strand annealing (SSA). Previously, a strategy called homology-independent targeted integration (HITI) was described to perform exogenous DNA integrations *in vivo* to correct *DMD* deletions.[18,19] Due to its design, HITI can only recruit NHEJ as a repair pathway to perform DNA insertions. The proposed strategy in this study, template-assisted sequence knockin (TASK), is capable of recruiting SSA in addition to NHEJ through the inclusion of large homology arms (HAs) in the donor template, increasing the efficiency of DNA integration events in non-dividing cells.[20] TASK requires the delivery of three components to a cell using two adeno-associated viruses (AAVs). Components include any single-vector compatible Cas nuclease, its respective single-guide RNA (sgRNA) targeting the genomic insertion site, and the TASK donor template containing the coding sequence to be inserted, flanked by HAs and corresponding sgRNA target sites (Figure 1A). In this study, we used the *Staphylococcus aureus* Cas9 (*Sa*Cas9) to target the insertion site in the *Dmd* gene and generate a DSB while simultaneously releasing the TASK donor template from the AAV genome. Integration of the TASK donor via the NHEJ pathway may lead to inverted knocking in of the template. This inverted product would regenerate the flanking sgRNA target sites, allowing for excision and additional attempts at correct integration. Recruitment of the SSA pathway is initiated by long-range 5′–3′ end resection of the HAs using cellular exonucleases, followed by complementary annealing of the homologous sequences. The integration process is complete once non-homologous flaps are removed and nicks are ligated. Productive integration of the donor template following NHEJ or SSA is terminal since it does not result in regeneration of the sgRNA target sites.

As a proof of concept, we utilized a patient-representative deletion of *Dmd* exons 52–54, which generates a frameshift, introducing a premature stop codon in exon 55, stopping dystrophin expression (Figure 1B). *Dmd Δ52-54* mice, which we previously reported to develop severe left ventricular hypertrophy, skeletal muscle dysfunction, and typical dystrophic histology, were used to test this strategy.[21] Here, we developed a TASK-based precise DNA integration strategy for the *in vivo* correction of the *Dmd Δ52-54* deletion. We assessed the therapeutic efficacy of the strategy by delivering the gene-editing components to muscle tissue both locally and systemically using a dual AAV9 delivery system. Systemic delivery was performed by temporal vein injections of neonatal P1-2 *Dmd Δ52-54* male pups. DNA, RNA, and protein analyses were performed to determine the level of correction in each tissue. Our strategy successfully restored therapeutically relevant levels of full-length dystrophin in the heart and in skeletal muscles, leading to significant improvements in muscle function, enhanced mobility, and overall healthier physiology.

TASK represents a significant improvement for restoring full-length dystrophin in a majority of the DMD patient population, particularly those with large deletions. With further refinement and development, this strategy could be expanded to treat a broader spectrum of the population affected by this disorder.

RESULTS

Correction strategy for the *Dmd Δ52-54* model of DMD using a TASK-based DNA knockin strategy

The *Dmd Δ52-54* mouse model exhibits a loss of dystrophin expression due to an out-of-frame deletion that introduces a premature stop codon in exon 55. Here, we have developed a TASK-based strategy to insert exons 52–54 into *Dmd* intron 54, restoring the complete dystrophin coding sequence. Our approach utilized a dual-vector system comprising one AAV vector that expressed the *Sa*Cas9 nuclease and sgRNA, called TASK-in54g3, and a second vector that carried the TASK donor template. The sequence of exons 52–54 was coded for in the donor template along with the native splice donor and acceptor sequences, HAs, and the TASK-in54g3 cut sites oriented in the opposite orientation from the target site at the genomic integration site. Once inside the myofiber nuclei, the *Sa*Cas9 liberates the donor template from the AAV genome while also generating a single DSB in intron 54. Following this, the SSA repair pathway can initiate, where exposed ends of the HAs are resected by cellular exonucleases, resulting in single-stranded DNA that can anneal to the complementary sequences at the integration site. The knockin process is complete once the backbone is sealed by DNA ligase. Alternatively, NHEJ is recruited instead and repairs the DSB by ligating the TASK donor template directly into the genome. Although resulting in structurally different integration products, both repair pathways restore the full-length dystrophin coding sequence.

Validation and optimization of the TASK-mediated exon 52–54 DNA knockin in mouse myotubes

To validate the targeted integration of the TASK donor sequence, we delivered the system to differentiated C2C12 myotubes using AAV-DJ vectors (Figure 1C). Following differentiation and transduction of the myotubes using the AAV-TASK system, genomic PCR was used to confirm integration of the donor template at the genomic level (Figure 1D). Additionally, PCR amplification and Sanger sequencing of cDNA generated from the treated cells revealed the presence of the integrated exons in the *Dmd* transcript, confirming proper splicing (Figure 1E). To further optimize the system in muscle cells, we generated additional donor templates with varying HA lengths. AAV-DJs carrying each donor were used to transduce the myotubes. Digital PCR (dPCR) analysis quantified integration efficiency at the genomic level, showing a positive correlation with HA length and peaking in constructs with 800-bp HAs (Figure 1F). To evaluate the specificity of the TASK-in54g3 sgRNA, we performed *in silico* off-target prediction followed by targeted sequencing of top-ranked off-target sites (Table S2). No significant off-target editing was detected by targeted Sanger sequencing above background levels (data not shown). These results validate our TASK-based strategy for targeted exon 52–54 integration in the *Dmd* gene.

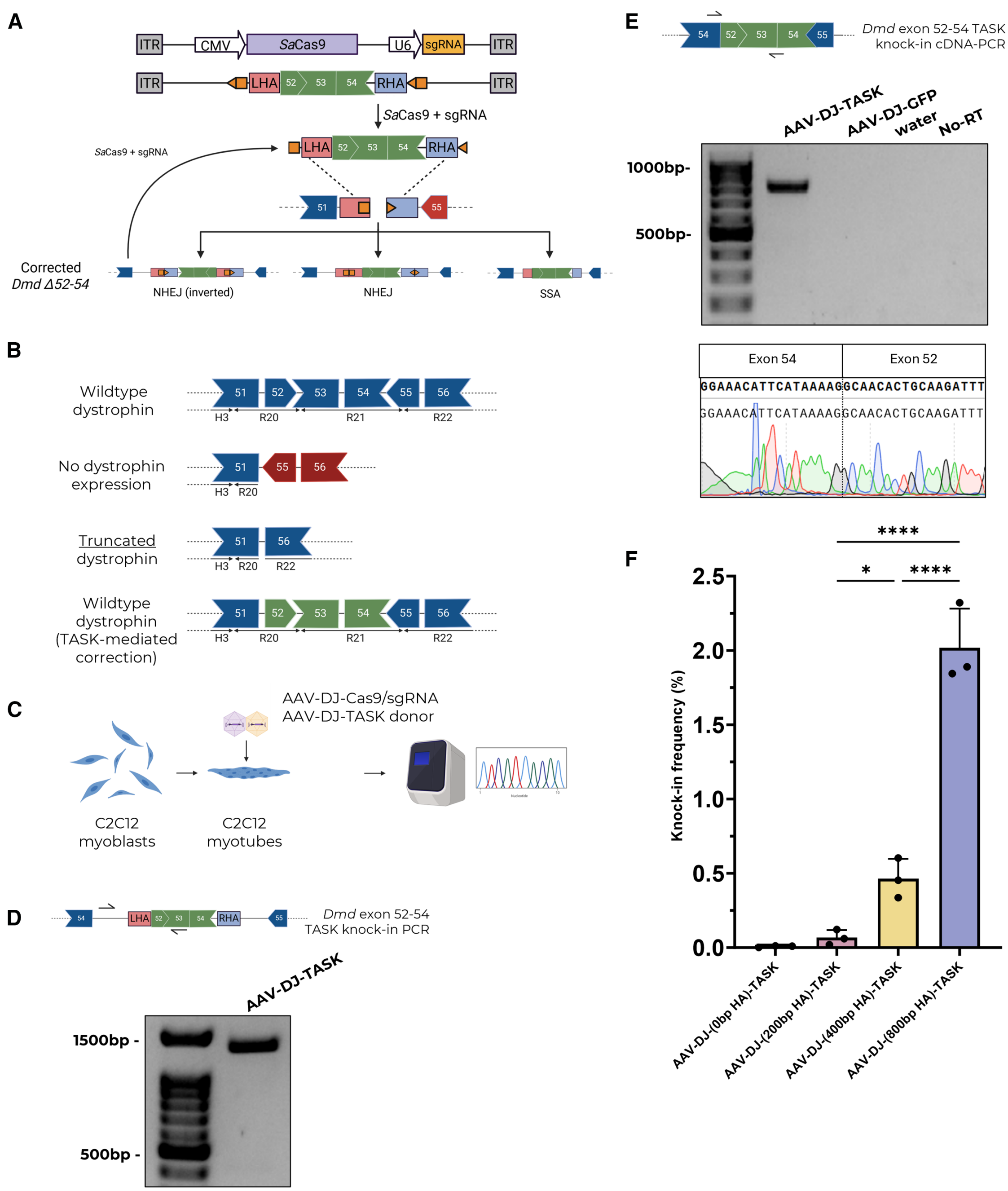

Figure 1. TASK-mediated knockin of *Dmd* exons 52–54 in differentiated mouse myotubes

(A) Schematic of dual AAV vector approach for TASK-mediated exon 52–54 integration; orange pentagon represents the sgRNA target sequence, divided up into the spacer sequence (orange square) and the protospacer adjacent motif (orange triangle). LHA and RHA correspond to left and right homology arms (HAs), respectively. (B) Schematic representation of dystrophin gene variants showing wild-type dystrophin, absence of dystrophin expression due to deletion of exons 52–54, truncated dystrophin expression following exon 55 excision, and wild-type dystrophin expression following TASK-mediated exon integration. (C) C2C12 myoblasts were differentiated into myotubes and edited with the AAV-DJ-TASK vectors. (D) Validation of SSA-mediated genomic knockin by PCR. (E) Validation of the exon 52–54 sequence incorporation in the *Dmd* transcript by cDNA PCR. (F) Optimization of TASK donor construct HA length in C2C12 myotubes using dPCR. One-way ANOVA, followed by Tukey's post hoc multiple comparisons test (*$p < 0.05$; **$p < 0.01$; ***$p < 0.001$; **** $p < 0.0001$; mean ± SD; $n = 3$ replicates).

Restoration of wild-type dystrophin *in vivo* using local intramuscular injections

To validate this system design *in vivo*, we co-delivered AAV9s carrying the TASK system into the tibialis anterior of 6-week-old male *Dmd Δ52-54* mice (Figure 2A). Tissues were harvested 3 weeks post-injection, and genomic-level PCR was used to confirm the SSA integration product (Figure 2B). The percentage of wild-type transcript restored in the treated tibialis anteriors averaged 6.40% ± 0.80% (normalized to *Dmd* exon 51), according to dPCR analysis (Figure 2C). Western blot and immunofluorescence analysis demonstrated restoration of the full-length dystrophin protein and confirmed its localization to the muscle cell sarcolemma (Figures 2D and S1). These results demonstrated the AAV9-TASK system's ability to integrate exons 52–54 into the *Dmd* gene, inducing full-length dystrophin protein expression *in vivo*.

Restoration of full-length dystrophin in skeletal and cardiac muscles via systemic delivery of the AAV9-TASK DNA knockin system

To assess the therapeutic efficacy of the TASK strategy in correcting the *Dmd Δ52-54* deletion, we delivered AAV9-TASK vectors systemically via the temporal vein to *Dmd Δ52-54* P1 male pups (Figure 3A). Skeletal and cardiac tissues were harvested for molecular analysis 12 weeks post-injection. The larger NHEJ and smaller SSA integration products were found in select tissues by PCR amplification of the genome-donor junction, indicating that the presence of HAs in the TASK donor template allows for knockins through both pathways (Figure 3B). Genomic-level dPCR analysis indicated the highest level of SSA-mediated editing in the heart, followed by skeletal tissue. The same pattern held true when quantifying the level of wild-type *Dmd* transcript using dPCR (Figures 3C and 3D). Heart cryosections stained for dystrophin and laminin-α2 showed that 12.99% ± 1.00% of fibers were corrected in treated animals (Figure 4A). Immunofluorescent staining of dystrophin revealed the presence of corrected fibers throughout all skeletal muscles. α-Sarcoglycan is a member of the DAPC and strongly localizes to the sarcolemma of skeletal muscles that express dystrophin. Staining serial sections of skeletal muscles for dystrophin and α-sarcoglycan reveals strong colocalization of the two proteins in corrected fibers, demonstrating that AAV9-TASK-mediated dystrophin restoration successfully recruits the DAPC (Figure 4B). Whole-protein lysates of cardiac and skeletal muscles were analyzed by western blotting, which verified the restoration of full-length dystrophin. The results indicated that restoration was highest in the heart at 19.94% ± 6.11%, while skeletal tissue showed moderate restoration levels at 0.79% ± 0.57% in the diaphragm, 0.63% ± 0.35% in the tibialis anterior, and 0.77% ± 0.49% in the triceps (Figure 4C). These results demonstrate that systemic delivery of the TASK system can restore full-length dystrophin expression in cardiac and skeletal tissues.

AAV9-TASK treatment of *Dmd Δ52-54* neonates reduces cardiac hypertrophy and improves function 12 weeks post-injection

Left ventricular hypertrophy is a defining phenotype of the *Dmd Δ52-54* model, and given the high efficiency of our system in correcting cardiomyocytes, we conducted echocardiography analysis on treated animals to quantify any improvements. We assessed heart rate, stroke volume, ejection fraction, and fractional shortening in systemically treated animals 12 weeks after injection, observing significant improvements in every parameter (Figures 5A and S2). To compare the progression of left ventricular hypertrophy in treated and untreated mice, we measured the thickening of the posterior and anterior left ventricular walls as well as the diameter of the left ventricle. Notably, treated animals showed less hypertrophy in the anterior and posterior ventricular walls, while left ventricular diameter significantly increased in tandem with this reduction (Figures 5B and 5C). These findings highlight that the restoration of full-length dystrophin not only reduces the progression of left ventricular hypertrophy at 12 weeks but it also dramatically enhances cardiac function, resulting in improved overall cardiac physiology in treated animals (Videos S1, S2, and S3). Quantification of the total fibrotic area from heart cryosections stained with Masson's trichrome revealed a significant increase in fibrosis in the AAV9-TASK-treated mice compared to the AAV9-GFP group (Figure S3). One possible explanation is an immune response triggered by Cas9 expression, leading to increased fibrosis.[22] Cas9 expression has been shown to elicit humoral and cytotoxic T cell responses, potentially contributing to inflammation-driven fibrosis in muscle tissues.[23] Further investigation is needed to determine whether the observed fibrosis is transient and whether immune modulation strategies could mitigate this response.

Restoration of full-length dystrophin via AAV9-TASK treatment of *Dmd Δ52-54* neonates improves skeletal muscle function and mobility

To evaluate whether the levels of dystrophin restoration in skeletal tissue were sufficient to yield significant improvements in muscle function, we conducted fatigue grip strength tests on the treated animals. The trials were performed back to back, with 5 min of rest in between; in the initial trial, there was no significant difference in grip strength between the TASK-treated animals and the untreated group. However, in the second trial, the TASK-treated mice demonstrated a 22.38% increase in grip strength (Figures 6B and S4). We also evaluated the performance of treated animals in the open-field test to determine whether the combined restoration of dystrophin in the skeletal and cardiac muscles was adequate to improve overall functional mobility. Significant improvement was seen in several parameters for the TASK-treated mice, including shorter resting times, longer travel distances, and increased frequency of rearing events (Figure 6C). The percentage of time spent in the center and the number of center entries showed encouraging trends as well, although they fell short of the threshold for statistical significance (Figure S5). Another notable observation was related to excess weight gain, often a consequence of muscle hypertrophy in both patients and mouse models of DMD. Upon comparing the weights of the TASK-treated animals to those of the untreated group, we observed a significant reduction in excess body weight in the TASK group. This suggests improved skeletal muscle function,

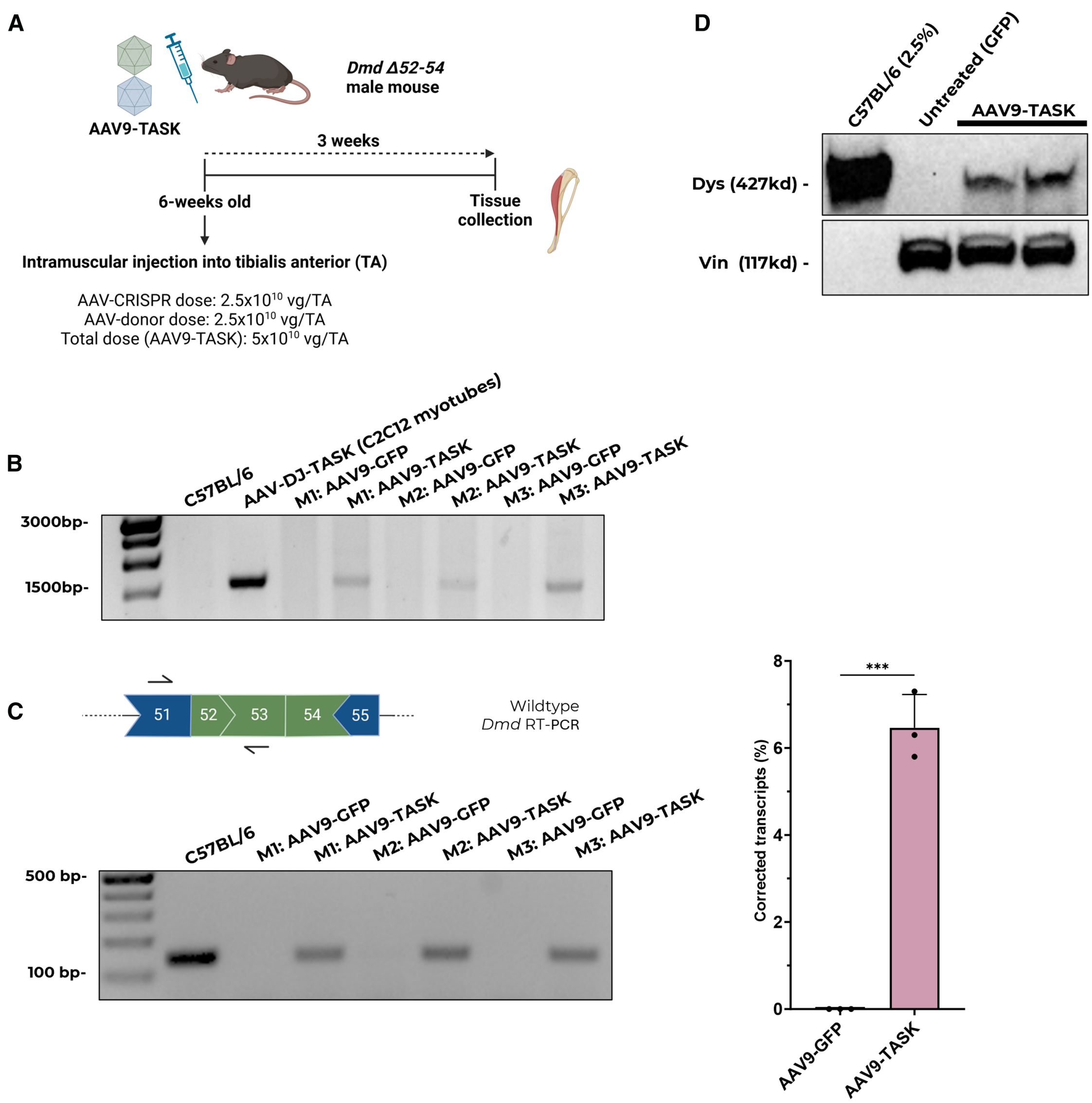

Figure 2. AAV9-TASK-mediated exon 52–54 integration restores full-length dystrophin in *Dmd Δ52-54* mouse tibialis anterior muscles

(A) We injected 6-week-old male *Dmd Δ52-54* mice in the tibialis anterior muscles with the AAV9-TASK (left leg) and AAV9-GFP (right leg) vectors. (B) Validation of SSA-mediated genomic knockin by DNA PCR. M1–M3 corresponds to the three treated mice. (C) Validation and quantification of wild-type *Dmd* transcript restoration by cDNA PCR and dPCR (expression of wild-type *Dmd* transcript normalized to *Dmd* exon 51), respectively. M1–M3 corresponds to the three treated mice. Unpaired Student's t test (*$p < 0.05$; **$p < 0.01$; ***$p < 0.001$; **** $p < 0.0001$; mean ± SD; $n = 3$ mice). (D) Western blot for dystrophin shows restoration of full-length protein expression. The C57BL/6 positive control sample was prepared by diluting C57BL/6 mouse tibialis anterior whole-protein lysate to 2.5% of its original concentration using ultrapure water.

reducing the need for compensatory hypertrophy (Figure 6A). Overall, the functional and behavioral test results highlight the systemic benefits of restoring full-length dystrophin through our TASK-based DNA knockin strategy, showing notable physiological improvements in the treated animals.

DISCUSSION

We previously attempted to correct the *Dmd Δ52-54* deletion *in vivo* by systemically treating the model with an AAV-CRISPR to excise exon 55. Although this approach restored the expression of dystrophin in skeletal muscles, it failed to yield functional improvements.[14] To further understand the therapeutic efficacy of the truncated dystrophin protein produced from the *Dmd Δ52-55* gene, we generated a murine model harboring this deletion. While these mice expressed the truncated dystrophin protein in all muscle cells, they still demonstrated exercise-induced skeletal muscle dysfunction, affirming that truncated dystrophin, even when ubiquitously expressed, does not adequately restore muscle function.[24] These outcomes highlight the limitations of strategies that bring about the expression of truncated dystrophin protein.

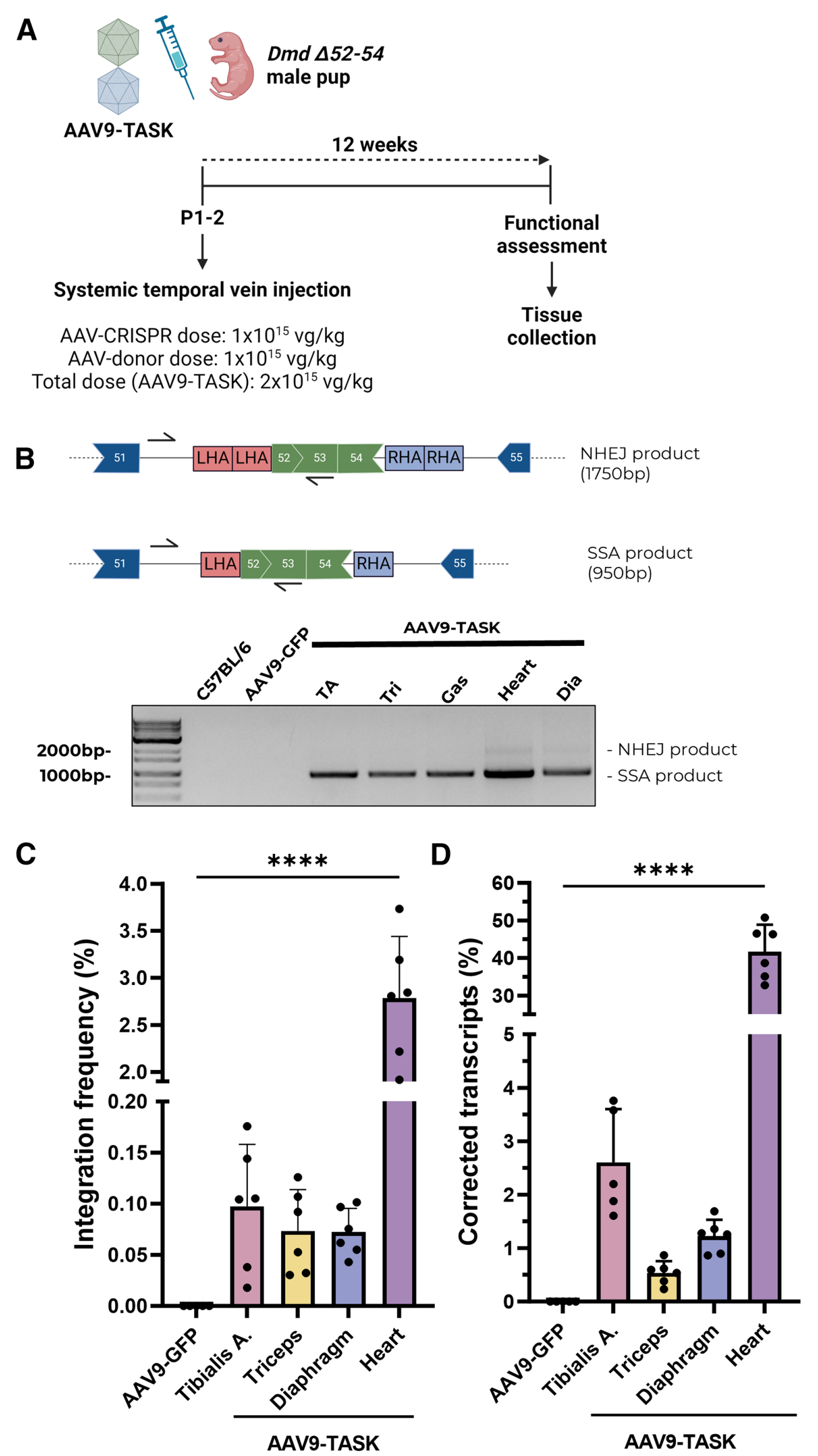

Figure 3. Systemic delivery of the AAV9-TASK restores full-length dystrophin expression in *Dmd Δ52-54* mouse skeletal and cardiac tissue

(A) P1 male *Dmd Δ52-54* neonates were injected through the temporal vein with AAV9-TASK or AAV9-GFP vectors and analyzed 12 weeks post-treatment. (B) Validation of both the NHEJ and SSA-mediated TASK-donor genomic integration products by PCR. (C) dPCR analysis of genomic DNA from muscles of treated animals indicates AAV9-TASK-mediated DNA integrations. The highest level of editing was achieved in the heart, followed by lower levels of integration in the skeletal tissues. (D) Transcript-level analysis using dPCR indicates restoration of wild-type *Dmd* transcript in skeletal and cardiac muscles of treated animals. Trends in genomic-level editing and transcript restoration match across the analyzed tissues. One-way ANOVA, followed by Dunnett's post hoc multiple comparisons test (*$p < 0.05$; **$p < 0.01$; ***$p < 0.001$; ****$p < 0.0001$; mean ± SD; $n = 5–6$ mice).

In contrast, TASK-mediated correction of the *Dmd Δ52-54* deletion *in vivo* directly addresses these critical limitations. TASK successfully restored the expression of full-length dystrophin protein, translating into functional improvements. We co-delivered AAV9 vectors carrying the TASK components to *Dmd Δ52-54* mice, systemically restoring the complete *Dmd* coding sequence. To achieve this, the TASK donor template was inserted using a targeted genomic DSB generated by *Sa*Cas9 and the endogenous NHEJ and SSA repair pathways. Molecular analysis of treated mice indicated restoration of full-length dystrophin in skeletal and cardiac tissue. Open-field and grip[25] strength testing, as well as echocardiography, fully evaluated the therapeutic effectiveness of the strategy. The outcomes of these tests demonstrated marked enhancements in heart and skeletal muscle function, underscoring the potential of TASK-mediated gene correction as an approach for treating DMD. It is important to note that although the *Dmd Δ52-54* model exhibits cardiac hypertrophy, patients predominantly present with dilated cardiomyopathy (DCM), and those that initially present with cardiac hypertrophy often undergo remodeling of the left ventricle, and progress to DCM.[26] The *Dmd Δ52-54* mice exhibit cardiac hypertrophy up to 52 weeks but may follow a similar trajectory later in life. If so, then it is crucial to evaluate the effect of full-length dystrophin restoration using TASK in this model to determine whether the observed improvements are sustained later into the natural history of the disease.

We observed a higher level of dystrophin restoration in the myocardium compared to skeletal muscle. This disparity can be primarily attributed to differences in AAV9 tropism and tissue vascularization, which allow for more efficient transduction of cardiac tissue.[27,28] Furthermore, the higher turnover rate of myofibers compared to cardiomyocytes may contribute to the reduced persistence of genomic edits in dystrophic skeletal muscles over time.[29] Long-term persistence of edits in myofibers is only possible through the correction of satellite cells.[30] Through asymmetric division, these cells give rise to myoblasts, which then aid in the repair and regeneration of damaged myofibers. Although TASK should be capable of editing satellite cells, AAV9s do not efficiently transduce these cell populations.[25] Novel myotropic vectors such as MyoAAV-1A have shown improved targeting of satellite cells compared to older serotypes; however, the absolute rate of transduction still remains lower than optimal.[31] Finally, differences in the cellular repair pathways available to myofibers and cardiomyocytes may explain the differences observed in editing efficiency of skeletal and cardiac tissues, although further investigation is needed to delineate the underlying mechanisms. This work represents the first application of an exogenous DNA knockin strategy that rescues functional deficiencies in skeletal and cardiac muscles in a deletion model of DMD. Therefore, the TASK-based approach offers substantial therapeutic advancements over previous methods by ensuring that dystrophin restoration extends beyond molecular correction and produces quantifiable functional improvements.

TASK is particularly well suited for the treatment of DMD, given that even very low levels of full-length dystrophin (<0.5%) can lead to milder disease phenotypes and prolonged ambulation in patients.[32–36] This highlights the potential of the AAV-TASK DNA knockin system, which achieved significant dystrophin restoration in *Dmd Δ52-54* mice and led to substantial improvements in both

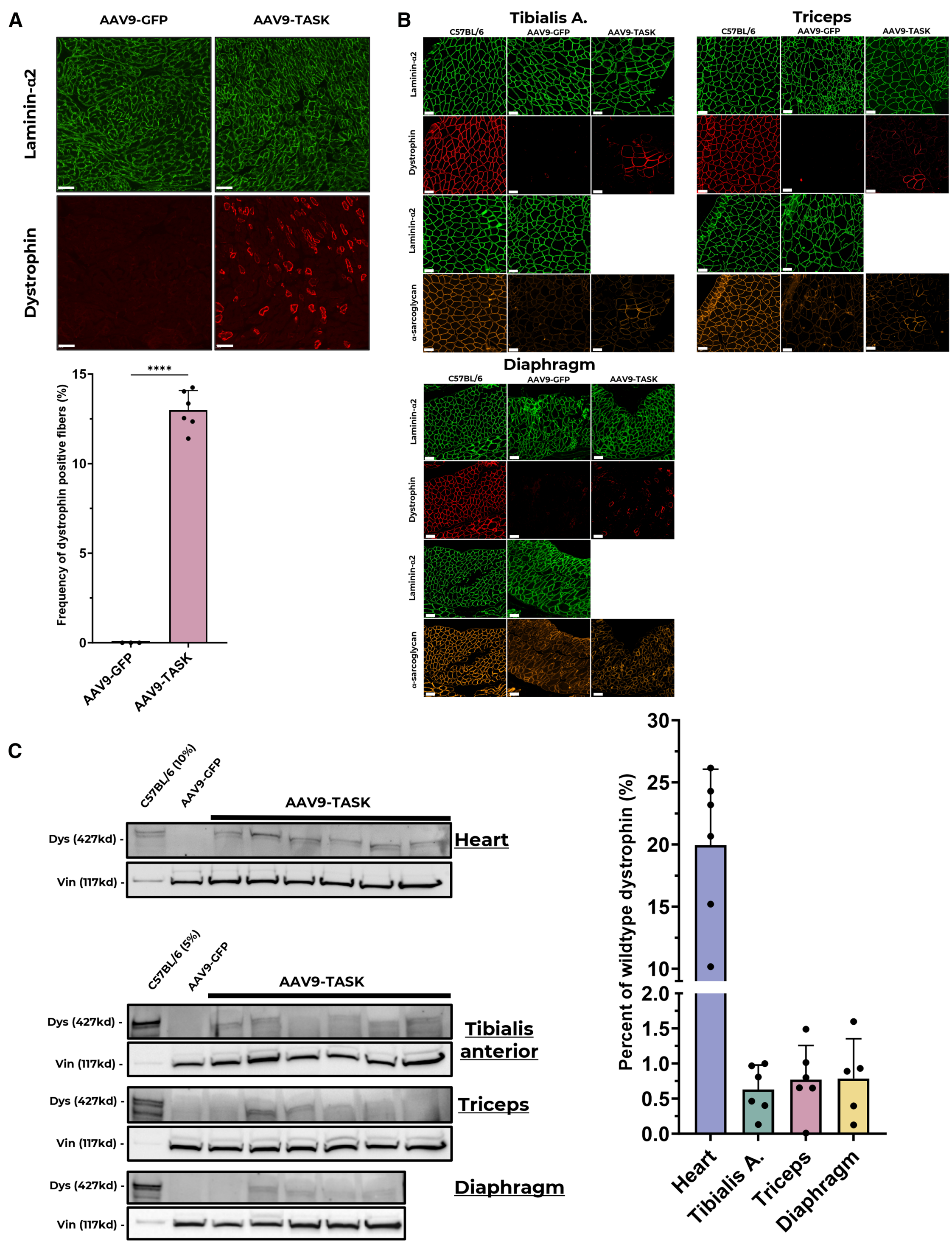

Figure 4. Immunofluorescent and western blot analysis indicates restoration of full-length dystrophin protein and recruitment of the DAPC in the muscles of treated animals

(A) Quantified dystrophin and laminin-α2 immunofluorescence staining reveals a significant increase in the number of dystrophin-positive fibers in the hearts of treated mice (scale bar, 50 μm; each dot represents the mean of 9–12 images per mouse). Unpaired Student's t test (*$p < 0.05$; **$p < 0.01$; ***$p < 0.001$; ****$p < 0.0001$; mean ± SD; $n = 6$

(legend continued on next page)

skeletal and cardiac muscle function. By administering early in the patient's life, this strategy may significantly enhance treatment outcomes, underscoring the efficacy of targeted gene-editing approaches in treating DMD. However, improving the efficiency and safety profile of this system is essential to its clinical translation.

Although AAV9 vectors are the current gold standard for delivering DNA cargo to muscle cells, they have several drawbacks, such as the requirement for high titers and off-target transduction of tissues, which complicate clinical applications. Novel myotropic vectors that improve muscle tropism and off-target transduction have been developed recently, potentially improving the delivery and effectiveness of gene-editing strategies such as TASK.[31,37,38] Although these vectors have not yet been approved for use in the clinic, they represent a promising avenue for improving the delivery, safety, and overall success of gene therapies. Additionally, the use of a muscle-specific promoter such as CK8e or Spc5-12 in place of the ubiquitous CMV promoter could improve tissue-targeted expression of genome editing components, reducing off-target effects and improving the safety profile of the TASK system for clinical translation. Furthermore, alternative approaches to knockins, called "gene writing," have been developed that enable the integration of longer DNA sequences than those currently done by HITI and TASK.[39,40] Nevertheless, the gene-editing components needed for these strategies are too large to be delivered by AAVs, demanding either the miniaturization of components or the use of alternative delivery vectors such as nanoparticles. The TASK-mediated DNA integration system shows great promise for progressing toward efficient genetic therapies, potentially addressing the major obstacles presented by diseases like DMD.

Similar to other individualized therapies, TASK shares the limitation of high design and manufacturing costs associated with gene-editing-based strategies for correcting patient-specific mutations. To correct any given deletion, TASK requires the redesign of both the sgRNA and the TASK repair template (insert and HAs). Delivery of the system using two AAV vectors is also not ideal since it requires the administration of much higher doses, risking a strong immune response. Single-AAV knockin strategies that package the Cas9, sgRNA, and repair template sequences in the same vector have been designed and tested.[41] However, packaging constraints prevent these vectors from carrying large HAs like those employed in this study. Additionally, a single-vector system could be safer due to the degradation of the AAV after liberation of the repair donor by cellular exonucleases. This inherent self-inactivation property prevents long-term expression of the Cas9 protein, improving the safety profile. With the discovery of smaller Cas proteins such as NanoCas,[42] designing efficient single-vector DNA knockin strategies can be a realistic option.

DNA insertion strategies such as the TASK-based system described here have the potential to correct large-scale genetic mutations and restore the wild-type forms of genes. This sets them apart from most gene-editing therapies, which mainly involve gene disruption, activation of compensatory factors, transient expression of therapeutic genes, or restoration of a truncated, partially functional gene product. Importantly, the restoration of full-length dystrophin is highly preferred when designing gene-editing-based correction strategies for DMD, as truncated forms of the protein may not fully restore functionality. This work expands the range of genome-editing tools that can be used in clinical settings to address the underlying causes of genetic disorders and highlights the therapeutic potential of the TASK-based DNA knockin strategy in correcting large, multi-exonic deletions, representative of most DMD patients.

MATERIALS AND METHODS

Experimental design

This study aimed to evaluate the efficacy of AAV9-TASK in restoring dystrophin expression in skeletal and cardiac muscle fibers. The experimental design included *in vitro* validation and optimization followed by systemic *in vivo* delivery to correct the *Dmd Δ52-54* deletion. Histological, molecular, and functional assessments were done 12 weeks post-injection. Analysis was performed at the DNA and RNA levels using dPCR followed by western blot and immunofluorescence analysis to quantify dystrophin protein restoration. Functional tests included echocardiography to quantify heart function, grip strength to measure skeletal muscle function, and open-field tests to measure overall mobility.

CRISPR sgRNAs guide design and cloning

The sgRNA (TASK-in54g3) utilized in this study was designed using https://www.benchling.com/ and selected based on minimal off-target activity. Complementary oligo strands were annealed, phosphorylated, and cloned into the pX601 plasmid for packaging into AAVs. sgRNAs utilized in this study are reported in Table S1.

AAV9 production

The pX601-*Sa*Cas9-TASK-in54g3, px601-TASK Donor-Template, and pX601-GFP plasmids for *in vivo* injections were packaged into AAV9 vectors and titered by the Boston Children's Hospital Viral Core. For *in vitro* experiments, the plasmids were packaged into AAV-DJs and titered by the Canadian Neurophotonics Viral Vector Core. All vectors were stored at -80°C.

C2C12 culturing, transduction with AAV-DJs, and DNA/RNA isolation

C2C12 myoblasts were maintained in Dulbecco's modified Eagle's medium (Wisent) supplemented with 10% (v/v) fetal bovine serum

mice). (B) Dystrophin, α-sarcoglycan, and laminin-α2 immunofluorescent staining show the presence of dystrophin-positive fibers as well as recruitment of the DAPC to the sarcolemma of corrected fibers in all skeletal muscles analyzed (scale bar, 50 μm). (C) Western blot quantification for dystrophin shows restoration of full-length protein expression in the heart and all skeletal muscles following systemic AAV9-TASK treatment. The C57BL/6 positive control samples were prepared by diluting the whole-protein lysates of the corresponding tissue in ultrapure water (*n* = 5–6 mice).

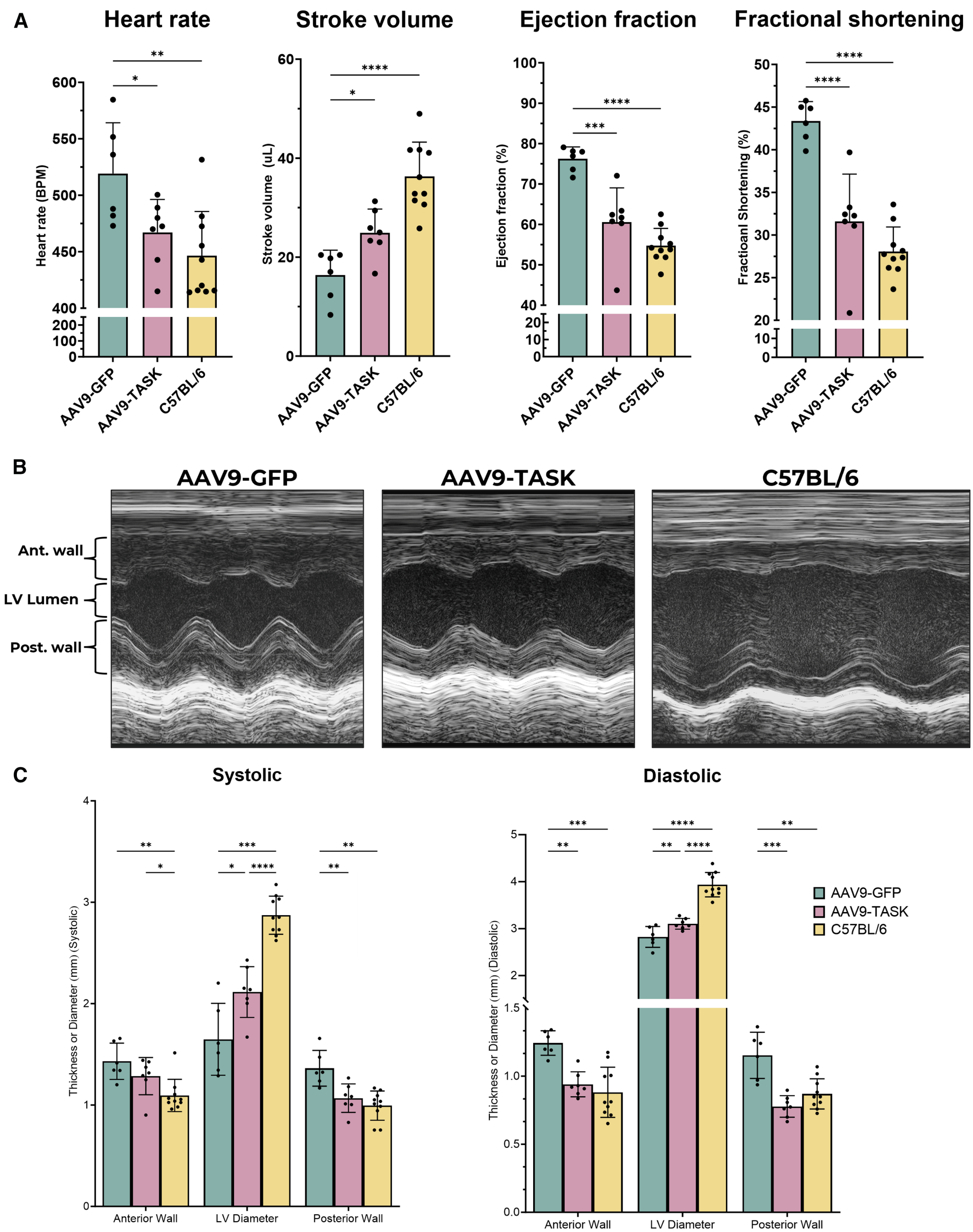

Figure 5. Systemic AAV9-TASK treatment of *Dmd Δ52-54* mice prevents left ventricular hypertrophy and improves cardiac function

(A) Heart rate, stroke volume, ejection fraction, and fractional shortening improved in TASK-treated mice 12 weeks post-injection. One-way ANOVA, followed by Dunnett's post hoc multiple comparisons test (*$p < 0.05$; **$p < 0.01$; ***$p < 0.001$; ****$p < 0.0001$; mean ± SEM; n = 6–10 mice). (B and C) Echocardiogram analysis of the left ventricle shows reduced hypertrophy in the posterior and anterior walls and an increase in ventricular diameter in treated animals when compared to untreated controls. Two-way ANOVA, followed by Tukey's post hoc multiple comparisons test (*$p < 0.05$; **$p < 0.01$; ***$p < 0.001$; ****$p < 0.0001$;; mean ± SEM; n = 6–10 mice).

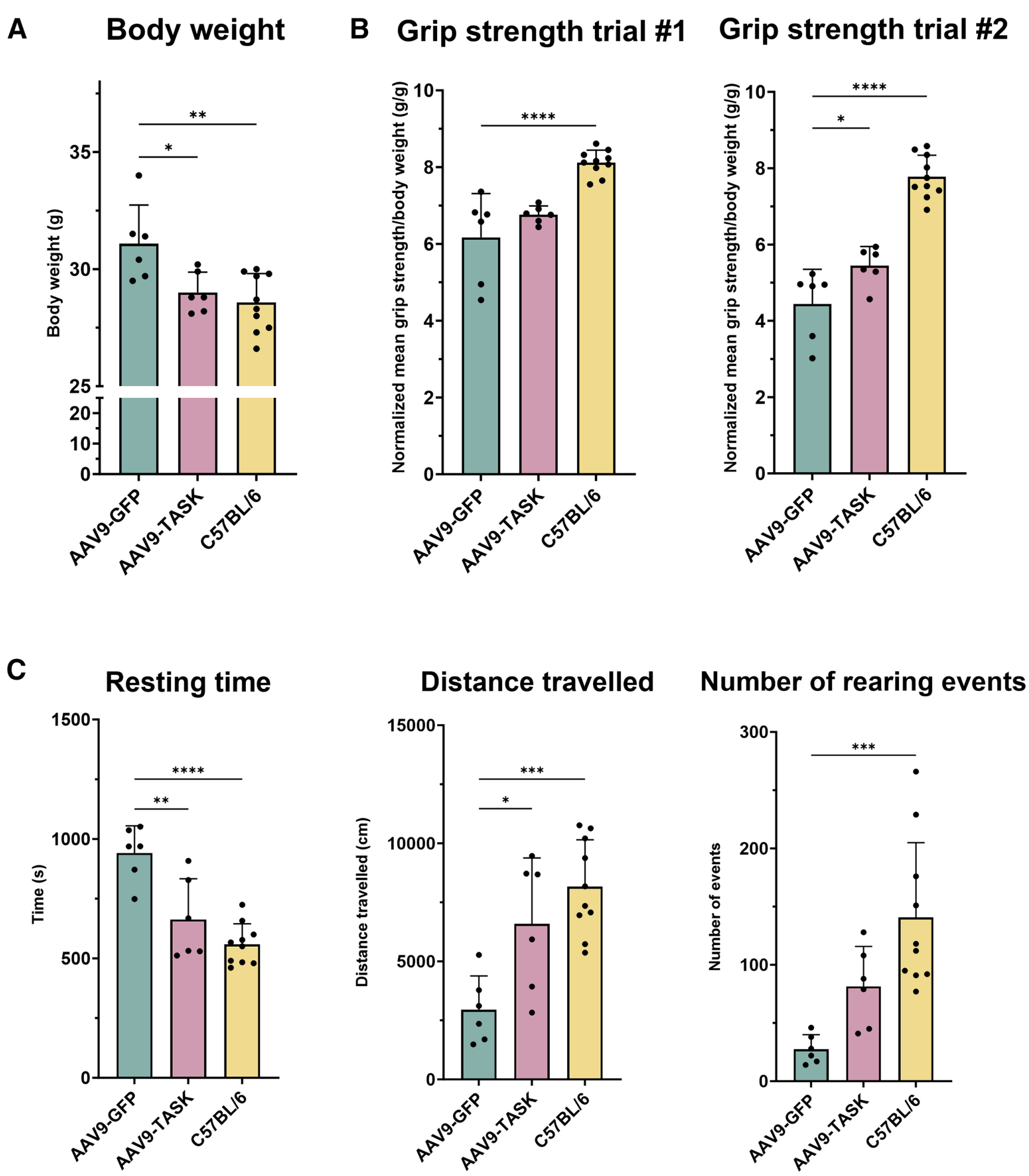

Figure 6. Significant improvements in skeletal muscle function and the mobility of AAV9-TASK-treated *Dmd Δ52-54* mice

(A) Body weight measurements of systemically treated and untreated animals, showing a reduction in muscle hypertrophy in AAV9-TASK-treated animals. (B) Forelimb and hindlimb grip strength test, showing improved skeletal muscle function in AAV9-TASK-treated animals. (C) Open-field test results indicate improved overall mobility in systemically treated *Dmd Δ52-54* mice. One-way ANOVA, followed by Dunnett's post hoc multiple comparisons test (*$p < 0.05$; **$p < 0.01$; ***$p < 0.001$; ****$p < 0.0001$; mean ± SEM; n = 6–10 mice).

(Wisent). For AAV-DJ transduction experiments, cells were seeded onto Matrigel (Corning)-coated 12-well plates at 50,000–60,000 cells per well. At 48 h after seeding, cells were washed with 1× PBS (Gibco) and switched to Dulbecco's modified Eagle's medium supplemented with 2% (v/v) horse serum (differentiation media), refreshed every 48 h until myotubes were fully formed (5–7 days). Transduction of myotubes was done at a dose of 5×10^9 viral genomes (vg)/well (2.5×10^9 of each TASK vector, 1:1 ratio). To transduce, the media was aspirated from the cells and replaced by 1 mL fresh differentiation media containing the AAV-DJs. Five days post-transduction, DNA and RNA were isolated directly from the wells using DirectPCR Lysis (Viagen) and TRIzol (Thermo Fisher Scientific) reagents following the manufacturers' protocols.

TASK-in54g3 *in vitro* off-target analysis

Mouse Neuro-2a (N2a) cells were maintained in Dulbecco's modified Eagle's medium (Wisent) supplemented with 10% (v/v) fetal bovine serum (Wisent). N2a cells were seeded (150,000–200,000/well) in a 12-well plate and transfected with the pX601 plasmid carrying the TASK-in54g3 guide using Lipofectamine 3000 (Thermo Fisher Scientific) 24 h post-seeding. Transfected cells were selected using 2 μg/mL puromycin for 4 days, and bulk genomic DNA was isolated using the Direct PCR solution (Viagen). Off-target regions were amplified using PCR, column purified, and Sanger sequenced using the SeqStudio Genetic Analyzers, according to manufacturers protocol. The off-target site sequences and primers that we used can be found in Table S2.

Animal husbandry

All mice were housed at The Centre for Phenogenomics (TCP) under the environmental regulation of a 12-h light/dark cycle with food and water provision in individual units (Techniplast). All animal procedures were conducted in compliance with the Animals for Research Act of Ontario and the Guidelines of the Canadian Council on Animal Care. Animal protocols performed at TCP were reviewed and approved by the local animal care committee.

Intramuscular injection of AAV9s

Before intramuscular injections, mice were anesthetized by isoflurane. Intramuscular injection of 6-week-old male *Dmd Δ52-54* mice was performed via slow longitudinal injection into tibialis anterior muscles using an ultrafine needle (31G) with a 40-μL solution of AAV9-GFP in the right leg or a prepared mixture of the dual AAV9-TASK vectors in the left leg, at a total dose of 5×10^{10} vg (1:1 ratio for the TASK vectors). Three weeks post-injection, the tibialis anteriors were dissected, coated with O.C.T. (VWR), and frozen in nitrogen-cooled isopentane (Sigma-Aldrich), followed by storage at −80°C as previously described.

Systemic injection of AAV9s

Mice were assigned to the AAV9-TASK and AAV9-GFP groups using a random selection process. To anesthetize mouse pups using hypothermia, the pup was placed on a cold surface lined with a paper towel or cloth, ensuring body contact with the cold for 2–4 min, or

until all movement stopped. For temporal vein injection of P1-2 neonatal male *Dmd Δ52-54* pups, a dose of 2.11×10^{12} viral genomes (1.055×10^{12} of each TASK vector, 1:1 ratio) was used. The injection volume was brought to 50 μL with 1× PBS. After the procedure, the pup was immediately warmed up on a heating pad and then returned to its mother. Functional tests were performed 12 weeks post-injection, and mice were euthanized by CO_2 inhalation. Tissues were dissected, coated with O.C.T., and frozen in nitrogen-cooled isopentane (Sigma-Aldrich), followed by storage at −80°C. Treatment group sizes were selected to maximize the delivered AAV dosage with the available viral titers.

Grip strength test

Forelimb and hindlimb grip strength tests were performed by The Centre for Phenogenomics in Toronto based on the TREAT-NMD SOP DMD_M.2.2.001 protocol. Age-matched 12-week-old male C57BL/6J and TASK/GFP-treated *Dmd Δ52-54* mice were lowered over the grid of the grip strength meter (Bioseb), with the torso parallel to the grid. Forepaws and hind paws were allowed to attach to the grid before pulling the mouse back by the tail, and the maximal grip strength value of the mouse was recorded. The test was done in triplicate, where the average grip strength value was corrected by the mouse's body weight. The test was also done as a fatigue grip strength test, where two trials were performed in total with 5 min of rest in between.

Echocardiography

For the echocardiography, male mice were scanned using the Vevo2100 ultrasound machine (VisualSonics) with a 30-MHz transducer as described previously. All mice were scanned under 1.5% isoflurane anesthesia for 20–30 min with careful monitoring of the body temperature to maintain it at 37°C–38°C (TREAT-NMD DMD_M.2.2.003). All measurements were conducted using the cardiac package of Vevo 2100 version 1.6.0 software.

Open-field test

For the open-field test, mice were placed in the frontal center of a transparent Plexiglas open field (41.25 × 41.25 × 31.25 cm) illuminated by 200 lx. A trained operator, unaware of the nature of the projects and treatments, performed the experiments. The VersaMax Animal Activity Monitoring System recorded vertical activities and total distance traveled for 20 min per animal.

DNA, RNA, and protein extraction

DNA from tissue was extracted using a DNAeasy Blood and Tissue Kit (Qiagen). A total of 40 ng of the yielded DNA was used for PCR with DreamTaq polymerase (Thermo Fisher Scientific); the primers are shown in Table S3. For RNA isolation, mouse tissues were sectioned, collected in 1.4-mm Zirconium Bead pre-filled tubes (OPS Diagnostics), and homogenized using a MagNA Lyser (Roche Diagnostics). RNA was then extracted using TRIzol reagent following the manufacturer's protocol. Protein was extracted from homogenized mouse tissue by adding a 1:1 solution of radioimmunoprecipitation assay (RIPA) homogenizing buffer (50 mM Tris-HCl pH 7.4, 150 mM NaCl, and 1 mM EDTA) and RIPA double-detergent buffer (2% deoxycholate, 2% NP-40, and 2% Triton X-100 in RIPA homogenizing buffer) supplemented with protease inhibitor cocktail (Roche). Protein concentration was quantified using a Pierce bicinchoninic acid protein assay kit (Thermo Fisher Scientific).

cDNA synthesis from RNA

We used 1 μg RNA for cDNA synthesis using the High-Capacity cDNA Reverse Transcription Kit reverse transcriptase (Applied Biosystems). The cDNA was used in subsequent PCRs or dPCRs, and the primers are shown in Tables S3 and S4.

dPCR assays

dPCR assays were done using the QuantStudio Absolute Q (Applied Biosystems) dPCR system. For analysis from genomic DNA, 100–200 ng sample was loaded into the reaction, and 1 μL cDNA was used for transcript analysis. All primers and probe sequences for the dPCR assays are shown in Table S4.

Tissue processing for histological and immunofluorescent staining

All harvested tissue was cryosectioned at 8 μm, mounted onto Superfrost Plus slides (VWR), and stored at −80°C.

Immunofluorescence staining and analysis

All muscle tissues were sectioned at 8 μm for immunofluorescence staining. Sections were fixed in ice-cold methanol and blocked with blocking buffer (3% normal goat serum and 0.2% BSA in PBS). Primary antibodies were incubated overnight at 4°C. The primary antibodies used were rabbit polyclonal anti-dystrophin (abcam15277, Abcam, 1:200), rat monoclonal anti-laminin-2 (α2 chain, 4H8-2, Sigma-Aldrich, 1:500), and rabbit monoclonal anti-α-sarcoglycan (MA5-37982, Thermo Fisher, 1:200). The secondary antibodies used were goat polyclonal anti-rat Alexa Fluor 594-conjugated (Thermo Fisher Scientific, 1:250) and goat polyclonal anti-rabbit Alexa Fluor 488-conjugated (Thermo Fisher Scientific, 1:250) antibodies. Slides were scanned with the PANNORAMIC 250 Flash III, and image acquisition was done on CaseViewer 2.4.

Masson's trichome staining and total fibrotic area quantification

Masson's trichrome staining was performed by the pathology laboratory of The Centre for Phenogenomics (TREAT-NMD SOP MDC1A_M.1.2.003). Slide scanning of samples was performed using the ZEISS Axioscan 7 Microscope Slide Scanner, image acquisition was performed on the ZEISS ZEN 3.9 software, and total fibrotic area quantification was performed on Fiji ImageJ software.

Western blotting

Whole-tissue isolate was prepared and western blotting was conducted according to the NuPAGE electrophoresis system (Thermo Fisher Scientific). The C57BL/6 positive control samples were prepared by diluting whole-protein lysates (respective for each tissue)

to the specified concentration. The primary antibodies utilized were mouse monoclonal anti-dystrophin (MANDYS8, Sigma-Aldrich, 1:5,000), mouse monoclonal anti-vinculin (V284, Millipore, 1:2,500), and mouse monoclonal anti-β-actin (sc-47778, Santa Cruz Biotechnology, 1:10,000). For heart isolate blots, immunofluorescent donkey anti-mouse immunoglobulin G (IgG) 647-nm secondary antibodies were used (Invitrogen, A32787). For skeletal muscle isolate blots, goat anti-mouse IgG horseradish peroxidase secondary antibodies were used (Abcam, ab205719) and developed with the Super Signal West Femto Maximum Sensitivity Substrate (Thermo Fisher Scientific, 34096). Western blot quantification was performed using densitometry in ImageLab, where dystrophin protein bands were normalized to vinculin and expressed as a percentage of wild-type levels present on the same blot.

Statistical analysis

All data were analyzed with 3–10 biological replicates and presented as mean ± SD. All p values were calculated by Student's t test and one- or two-way ANOVA with Tukey's or Dunnett's post hoc tests ($\alpha = 0.05$). GraphPad Prism version 10 was used to conduct the statistical analysis.

DATA AVAILABILITY

All data needed to evaluate the conclusions in the paper are present in the paper and/or the supplemental information. Additional data related to this paper may be requested from the authors.

ACKNOWLEDGMENTS

We acknowledge the members of the Cohn and Ivakine laboratories for their input and technical support in this study. We thank I. Vukobradovic and Vivian Bradaschia (Clinical Phenotyping and Histopathology Cores, The Centre for Phenogenomics) for their input. We thank Dr. K. Lau and P. Paroutis (SickKids Imaging Facility, SickKids Research Institute) for their input and technical support. We thank Dr. T. Paton and G. Casallo, as well as the Centre for Applied Genomics, for their input and technical assistance. Schematics were created with BioRender.com. We would like to thank the Canadian Institute of Health (grant no. 6210100686) and Jesse's Journey Foundation (grant no. 6100100206) for supporting this work.

AUTHOR CONTRIBUTIONS

S.F., M.J.R., R.M.M., E.K., E.A.I., and R.D.C. contributed to the conceptualization of the study. Methodology was designed and implemented by S.F. Investigation was carried out by S.F., R.M.M., E.K., M.J.R., E.H., N.K., H.A.T., L.C., and B.Y. Visualization and data presentation were performed by S.F., R.M.M., E.K., E.H., and N.K. Funding acquisition was secured by R.D.C. and E.A.I. Project administration was managed by S.F., E.A.I., and R.D.C., while supervision was provided by E.A.I. and R.D.C. The original draft of the manuscript was written by S.F., with review and editing contributions from S.F., R.M. M., E.K., E.A.I., and R.D.C.

DECLARATION OF INTERESTS

The authors declare no competing interests.

SUPPLEMENTAL INFORMATION

Supplemental information can be found online at https://doi.org/10.1016/j.ymthe.2025.05.005.

REFERENCES

1. Mendell, J.R., Shilling, C., Leslie, N.D., Flanigan, K.M., al-Dahhak, R., Gastier-Foster, J., Kneile, K., Dunn, D.M., Duval, B., Aoyagi, A., et al. (2012). Evidence-based path to newborn screening for Duchenne muscular dystrophy. Ann. Neurol. *71*, 304–313.
2. Ryder, S., Leadley, R.M., Armstrong, N., Westwood, M., de Kock, S., Butt, T., Jain, M., and Kleijnen, J. (2017). The burden, epidemiology, costs and treatment for Duchenne muscular dystrophy: an evidence review. Orphanet J. Rare Dis. *12*, 79.
3. Mah, J.K., Korngut, L., Dykeman, J., Day, L., Pringsheim, T., and Jette, N. (2014). A systematic review and meta-analysis on the epidemiology of Duchenne and Becker muscular dystrophy. Neuromuscul. Disord. *24*, 482–491.
4. Alfano, L.N., Charleston, J.S., Connolly, A.M., Cripe, L., Donoghue, C., Dracker, R., Dworzak, J., Eliopoulos, H., Frank, D.E., Lewis, S., et al. (2019). Long-term treatment with eteplirsen in nonambulatory patients with Duchenne muscular dystrophy. Medicine *98*, e15858.
5. Mendell, J.R., Goemans, N., Lowes, L.P., Alfano, L.N., Berry, K., Shao, J., Kaye, E.M., and Mercuri, E.; Eteplirsen Study Group and Telethon Foundation DMD Italian Network (2016). Longitudinal effect of eteplirsen versus historical control on ambulation in Duchenne muscular dystrophy. Ann. Neurol. *79*, 257–271.
6. Frank, D.E., Schnell, F.J., Akana, C., El-Husayni, S.H., Desjardins, C.A., Morgan, J., Charleston, J.S., Sardone, V., Domingos, J., Dickson, G., et al. (2020). Increased dystrophin production with golodirsen in patients with Duchenne muscular dystrophy. Neurology *94*, e2270–e2282.
7. Roshmi, R.R., and Yokota, T. (2019). Viltolarsen for the treatment of Duchenne muscular dystrophy. Drugs Today *55*, 627–639.
8. Mendell, J.R., Sahenk, Z., Lehman, K., Nease, C., Lowes, L.P., Miller, N.F., Iammarino, M.A., Alfano, L.N., Nicholl, A., Al-Zaidy, S., et al. (2020). Assessment of systemic delivery of rAAVrh74.MHCK7.Micro-dystrophin in children with Duchenne muscular dystrophy: A nonrandomized controlled trial. JAMA Neurol. *77*, 1122–1131.
9. Verhaart, I.E.C., and Aartsma-Rus, A. (2019). Therapeutic developments for Duchenne muscular dystrophy. Nat. Rev. Neurol. *15*, 373–386.
10. FDA. Expands Approval of Gene Therapy for Patients with Duchenne Muscular Dystrophy. https://www.fda.gov/news-events/press-announcements/fda-expands-approval-gene-therapy-patients-duchenne-muscular-dystrophy#:∼:text=Today%2C%20the%20U.S.%20Food%20and,mutation%20in%20the%20DMD%20gene.
11. Magri, F., Govoni, A., D'Angelo, M.G., Del Bo, R., Ghezzi, S., Sandra, G., Turconi, A. C., Sciacco, M., Ciscato, P., Bordoni, A., et al. (2011). Genotype and phenotype characterization in a large dystrophinopathic cohort with extended follow-up. J. Neurol. *258*, 1610–1623.
12. Aartsma-Rus, A., Van Deutekom, J.C.T., Fokkema, I.F., Van Ommen, G.-J.B., and Den Dunnen, J.T. (2006). Entries in the Leiden Duchenne muscular dystrophy mutation database: an overview of mutation types and paradoxical cases that confirm the reading-frame rule. Muscle Nerve *34*, 135–144.
13. Lim, K.R.Q., Maruyama, R., and Yokota, T. (2017). Eteplirsen in the treatment of Duchenne muscular dystrophy. Drug Des. Devel. Ther. *11*, 533–545.
14. Rok, M., Wong, T.W.Y., Maino, E., Ahmed, A., Yang, G., Hyatt, E., Lindsay, K., Fatehi, S., Marks, R., Delgado-Olguín, P., et al. (2023). Prevention of early-onset cardiomyopathy in Dmd exon 52–54 deletion mice by CRISPR-Cas9-mediated exon skipping. Mol. Ther. Methods Clin. Dev. *30*, 246–258.
15. Chemello, F., Chai, A.C., Li, H., Rodriguez-Caycedo, C., Sanchez-Ortiz, E., Atmanli, A., Mireault, A.A., Liu, N., Bassel-Duby, R., and Olson, E.N. (2021). Precise correction of Duchenne muscular dystrophy exon deletion mutations by base and prime editing. Sci. Adv. *7*, eabg4910. https://doi.org/10.1126/sciadv.abg4910.
16. Zhang, Y., Nishiyama, T., Li, H., Huang, J., Atmanli, A., Sanchez-Ortiz, E., Wang, Z., Mireault, A.A., Mammen, P.P.A., Bassel-Duby, R., and Olson, E.N. (2021). A consolidated AAV system for single-cut CRISPR correction of a common Duchenne muscular dystrophy mutation. Mol. Ther. Methods Clin. Dev. *22*, 122–132.
17. Min, Y.-L., Li, H., Rodriguez-Caycedo, C., Mireault, A.A., Huang, J., Shelton, J.M., McAnally, J.R., Amoasii, L., Mammen, P.P.A., Bassel-Duby, R., and Olson, E.N. (2019). CRISPR-Cas9 corrects Duchenne muscular dystrophy exon 44 deletion mutations in mice and human cells. Sci. Adv. *5*, eaav4324.
18. Pickar-Oliver, A., Gough, V., Bohning, J.D., Liu, S., Robinson-Hamm, J.N., Daniels, H., Majoros, W.H., Devlin, G., Asokan, A., and Gersbach, C.A. (2021). Full-length dystrophin restoration via targeted exon integration by AAV-CRISPR in a humanized mouse model of Duchenne muscular dystrophy. Mol. Ther. *29*, 3243–3257.

19. Stephenson, A.A., Nicolau, S., Vetter, T.A., Dufresne, G.P., Frair, E.C., Sarff, J.E., Wheeler, G.L., Kelly, B.J., White, P., and Flanigan, K.M. (2023). CRISPR-Cas9 homology-independent targeted integration of exons 1-19 restores full-length dystrophin in mice. Mol. Ther. Methods Clin. Dev. *30*, 486–499.

20. Yao, X., Wang, X., Hu, X., Liu, Z., Liu, J., Zhou, H., Shen, X., Wei, Y., Huang, Z., Ying, W., et al. (2017). Homology-mediated end joining-based targeted integration using CRISPR/Cas9. Cell Res. *27*, 801–814.

21. Wong, T.W.Y., Ahmed, A., Yang, G., Maino, E., Steiman, S., Hyatt, E., Chan, P., Lindsay, K., Wong, N., Golebiowski, D., et al. (2020). A novel mouse model of Duchenne muscular dystrophy carrying a multi-exonic Dmd deletion exhibits progressive muscular dystrophy and early-onset cardiomyopathy. Dis. Model. Mech. *13*, dmm045369. https://doi.org/10.1242/dmm.045369.

22. Dowling, J.J. (2022). CRISPR editing as a therapeutic strategy for Duchenne muscular dystrophy-anti-Cas9 immune response casts its shadow over safety and efficacy. Gene Ther. *29*, 575–577.

23. Hakim, C.H., Kumar, S.R.P., Pérez-López, D.O., Wasala, N.B., Zhang, D., Yue, Y., Teixeira, J., Pan, X., Zhang, K., Million, E.D., et al. (2021). Cas9-specific immune responses compromise local and systemic AAV CRISPR therapy in multiple dystrophic canine models. Nat. Commun. *12*, 1–12.

24. Perillat, L.O.M., Wong, T.W.Y., Maino, E., Ahmed, A., Scott, O., Hyatt, E., Delgado-Olguin, P., Ivakine, E.A., and Cohn, R.D. (2023). Generation and characterization of a novel mouse model of Becker Muscular Dystrophy with a deletion of exons 52 to 55. Preprint at bioRxiv. https://doi.org/10.1101/2023.11.16.567440.

25. Arnett, A.L., Konieczny, P., Ramos, J.N., Hall, J., Odom, G., Yablonka-Reuveni, Z., Chamberlain, J.R., and Chamberlain, J.S. (2014). Adeno-associated viral (AAV) vectors do not efficiently target muscle satellite cells. Mol. Ther. Methods Clin. Dev. *1*, 14038.

26. Harris, K.M., Spirito, P., Maron, M.S., Zenovich, A.G., Formisano, F., Lesser, J.R., Mackey-Bojack, S., Manning, W.J., Udelson, J.E., and Maron, B.J. (2006). Prevalence, clinical profile, and significance of left ventricular remodeling in the end-stage phase of hypertrophic cardiomyopathy. Circulation *114*, 216–225.

27. Zincarelli, C., Soltys, S., Rengo, G., and Rabinowitz, J.E. (2008). Analysis of AAV serotypes 1-9 mediated gene expression and tropism in mice after systemic injection. Mol. Ther. *16*, 1073–1080.

28. Duan, D. (2016). Systemic delivery of adeno-associated viral vectors. Curr. Opin. Virol. *21*, 16–25.

29. Chargé, S.B.P., and Rudnicki, M.A. (2004). Cellular and molecular regulation of muscle regeneration. Physiol. Rev. *84*, 209–238.

30. Yin, H., Price, F., and Rudnicki, M.A. (2013). Satellite cells and the muscle stem cell niche. Physiol. Rev. *93*, 23–67.

31. Tabebordbar, M., Lagerborg, K.A., Stanton, A., King, E.M., Ye, S., Tellez, L., Krunnfusz, A., Tavakoli, S., Widrick, J.J., Messemer, K.A., et al. (2021). Directed evolution of a family of AAV capsid variants enabling potent muscle-directed gene delivery across species. Cell *184*, 4919–4938.e22.

32. Godfrey, C., Muses, S., McClorey, G., Wells, K.E., Coursindel, T., Terry, R.L., Betts, C., Hammond, S., O'Donovan, L., Hildyard, J., et al. (2015). How much dystrophin is enough: the physiological consequences of different levels of dystrophin in the mdx mouse. Hum. Mol. Genet. *24*, 4225–4237.

33. van Putten, M., Hulsker, M., Nadarajah, V.D., van Heiningen, S.H., van Huizen, E., van Iterson, M., Admiraal, P., Messemaker, T., den Dunnen, J.T., 't Hoen, P.A.C., and Aartsma-Rus, A. (2012). The effects of low levels of dystrophin on mouse muscle function and pathology. PLoS One *7*, e31937.

34. van Putten, M., van der Pijl, E.M., Hulsker, M., Verhaart, I.E.C., Nadarajah, V.D., van der Weerd, L., and Aartsma-Rus, A. (2014). Low dystrophin levels in heart can delay heart failure in mdx mice. J. Mol. Cell. Cardiol. *69*, 17–23.

35. van Putten, M., Hulsker, M., Young, C., Nadarajah, V.D., Heemskerk, H., van der Weerd, L., 't Hoen, P.A.C., van Ommen, G.-J.B., and Aartsma-Rus, A.M. (2013). Low dystrophin levels increase survival and improve muscle pathology and function in dystrophin/utrophin double-knockout mice. FASEB J. *27*, 2484–2495.

36. de Feraudy, Y., Ben Yaou, R., Wahbi, K., Stalens, C., Stantzou, A., Laugel, V., Desguerre, I., FILNEMUS Network, Servais, L., Leturcq, F., and Amthor, H. (2021). Very low residual dystrophin quantity is associated with milder dystrophinopathy. Ann. Neurol. *89*, 280–292.

37. Weinmann, J., Weis, S., Sippel, J., Tulalamba, W., Remes, A., El Andari, J., Herrmann, A.-K., Pham, Q.H., Borowski, C., Hille, S., et al. (2020). Identification of a myotropic AAV by massively parallel *in vivo* evaluation of barcoded capsid variants. Nat. Commun. *11*, 5432.

38. El Andari, J., Renaud-Gabardos, E., Tulalamba, W., Weinmann, J., Mangin, L., Pham, Q.H., Hille, S., Bennett, A., Attebi, E., Bourges, E., et al. (2022). Semirational bioengineering of AAV vectors with increased potency and specificity for systemic gene therapy of muscle disorders. Sci. Adv. *8*, eabn4704.

39. Pandey, S., Gao, X.D., Krasnow, N.A., McElroy, A., Tao, Y.A., Duby, J.E., Steinbeck, B.J., McCreary, J., Pierce, S.E., Tolar, J., et al. (2025). Efficient site-specific integration of large genes in mammalian cells via continuously evolved recombinases and prime editing. Nat. Biomed. Eng. *9*, 22–39. https://doi.org/10.1038/s41551-024-01227-1.

40. Yarnall, M.T.N., Ioannidi, E.I., Schmitt-Ulms, C., Krajeski, R.N., Lim, J., Villiger, L., Zhou, W., Jiang, K., Garushyants, S.K., Roberts, N., et al. (2023). Drag-and-drop genome insertion of large sequences without double-strand DNA cleavage using CRISPR-directed integrases. Nat. Biotechnol. *41*, 500–512.

41. Ibraheim, R., Tai, P.W.L., Mir, A., Javeed, N., Wang, J., Rodríguez, T.C., Namkung, S., Nelson, S., Khokhar, E.S., Mintzer, E., et al. (2021). Self-inactivating, all-in-one AAV vectors for precision Cas9 genome editing via homology-directed repair *in vivo*. Nat. Commun. *12*, 6267.

42. Rauch, B.J., DeLoughery, A., Sper, R., Chen, S., Yunanda, S., Masnaghetti, M., Chai, N., Lin, J.C., Neckelmann, A., Bjornson, Y., et al. (2025). Single-AAV CRISPR editing of skeletal muscle in non-human primates with NanoCas, an ultracompact nuclease. Preprint at bioRxiv. https://doi.org/10.1101/2025.01.29.635576.

YMTHE, Volume *33*

Supplemental Information

Template-assisted sequence knockin rescues skeletal and cardiac muscle function in a deletion model of Duchenne muscular dystrophy

Sina Fatehi, Matthew J. Rok, Ryan M. Marks, Emily Huynh, Natalie Kozman, Hong Anh Truong, Lijun Chi, Bei Yan, Enzhe Khazeeva, Paul Delgado-Olguin, Evgueni A. Ivakine, and Ronald D. Cohn

Table S1. sgRNA sequence for the TASK system

sgRNA name	Spacer sequence (5'-3')	PAM sequence (5'-3')
TASK-in54g3	CATGATCTTGGCATATGGAG	TGGAGT

Table S2. TASK-in54g3 top off target sites and PCR/sequencing primers. Forward primer was used in the Sanger sequencing reaction.

**Sequence**	**Sequence (5'-3')**	**PAM sequence (5'-3')**
OT1 protospacer sequence	CATGAGCTATGCATATGGAG	ATGAAT
**Forward primer**	**TGCTGAGTGTAAGCAGCTATC**	
Reverse primer	TTCAAGCACAGAGCACACTC	
OT2 protospacer sequence	CATGTTCTGGGCATCTGGAG	GGGAGT
**Forward primer**	**ACCTGAGATGCAGTCTGTTGAG**	
Reverse primer	TCAAGTCCTTGGGACTGTTGG	
OT3 protospacer sequence	CAAGATCCTGGCATATGGGG	GTGGGT
**Forward primer**	**GTCTTCCCTCATCCACACCC**	
Reverse primer	ACCTCCATACACACATGCTGG	

Table S3. Genomic and cDNA PCR primer designs

Purpose	Primer name	Primer Sequence (5'-3')
Amplify the TASK-mediated *Dmd* exon 52-54 SSA integration product from treated C2C12 cells and muscle tissue following intramuscular injections in *Dmd Δ52-54* mice.	Dmd_d52-54_gKI_F	GAG ATT GGG TTC ACA GAA GGC
	Dmd_d52-54_gKI_R	TGT CCT ATG ACC TGT TCG GC
Amplify the edited transcript, containing an extra copy of exons 52-54 in TASK treated C2C12 cells.	Dmd_Ex52-54_int_F2	CTTGGGGAAACATTCATAAAAGGC
	Dmd_Ex52-54_int_R2	TTGTAACAGGGGTGCTTCATCC
Amplify the wildtype *Dmd* transcript, following intramuscular injections in *Dmd Δ52-54* mice.	mus_Dmd_ex51-F2	TGG GTG ATC TGG AAG ACA TCA ATG
	m_DMD_qPCR_ex52-R	GTT CTA GCT TCT TGA TTG CTG GTT TTG
Amplify the TASK-mediated *Dmd* exon 52-54 SSA and NHEJ integration products from muscle tissue following systemic injections in *Dmd Δ52-54* mice.	HMEJ-d52-54-KI Fw #2	ATTCCGTGTCTGTCTGTGCAT
	HMEJ-d52-54-KI RV #2	GGGCGTCTCTGTTCCAAATC

Table S4. dPCR assay primer and probe designs

Purpose	Primer/probe name	Primer/probe sequence (5'-3')
Assay to quantify wildtype *Dmd* transcripts.	CDXGRUV_F	CACAGAAACCAAGCAGTTGGC
	CDXGRUV_R	TCTTCCAAAGCAGCCTCTTGC
	CDXGRUV_P (probe)	ACGTGGCAAATGAT
Assay to quantify TASK integration events from genomic DNA.	HMEJ_del52-54_KI_ddPCR_F	GCTAAGACAGCATTACATTGA
	HMEJ_del52-54_KI_ddPCR_R	TTAAACCCGGCAAAAGCATA
	HMEJ_del52-54_Ki_ddPCR_Probe (probe)	CACAGATAATAGAGGACTTC
Reference primer/probe for both the assays listed above	ddpcr_m_ex51_F	TGCCATCTTCTTTGCTGTTG
	ddpcr_m_ex51_R	GTCCAAGCTCGGTTGAAGTC
	APGZHP9_V VIC (probe)	TACCTGCACTGGCAGAC

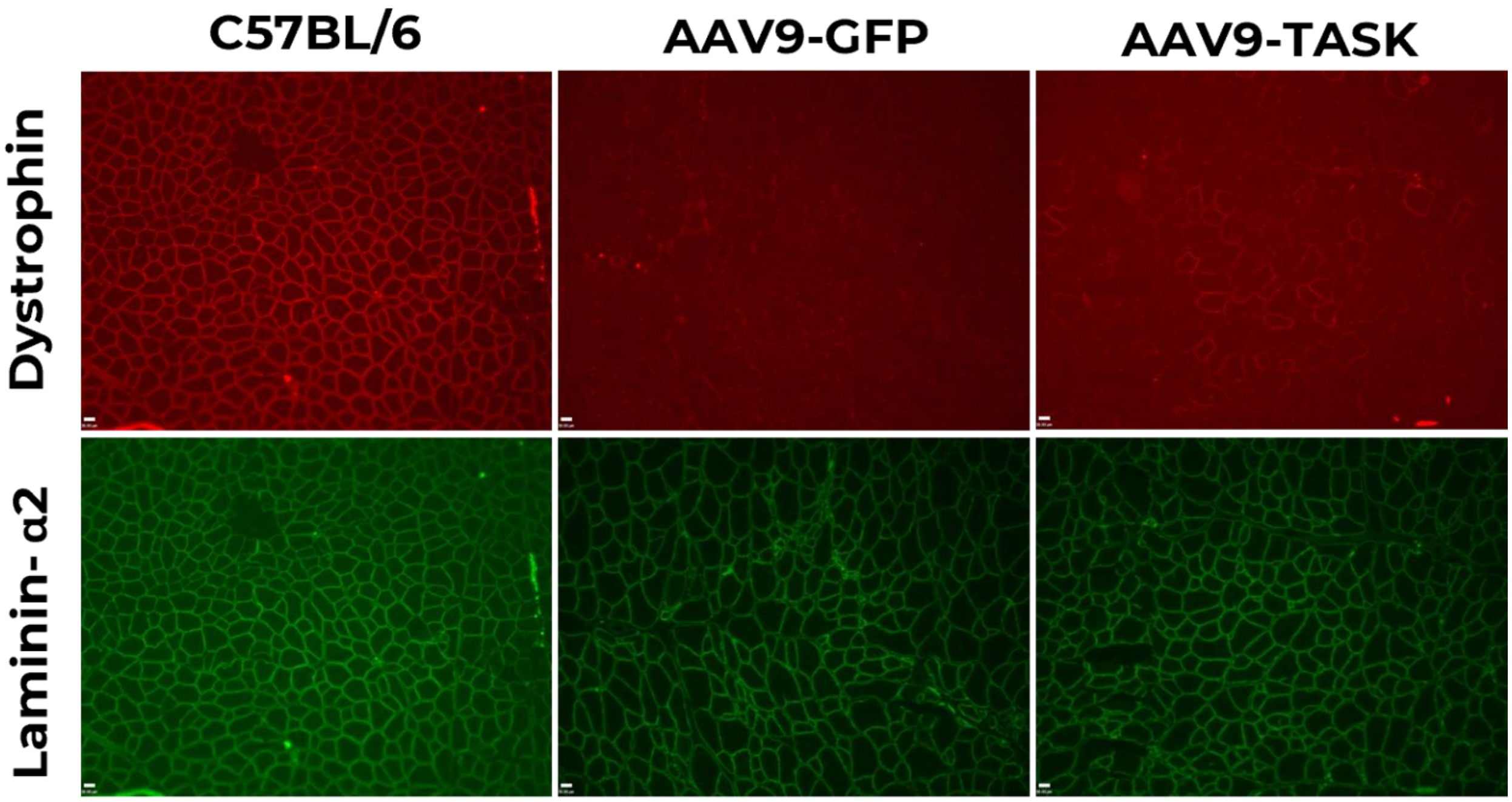

Figure S1: Dystrophin and laminin-α2 immunofluorescence staining shows the presence of dystrophin-positive fibers in the tibialis anterior of treated mice (scale bar, 30 µm).

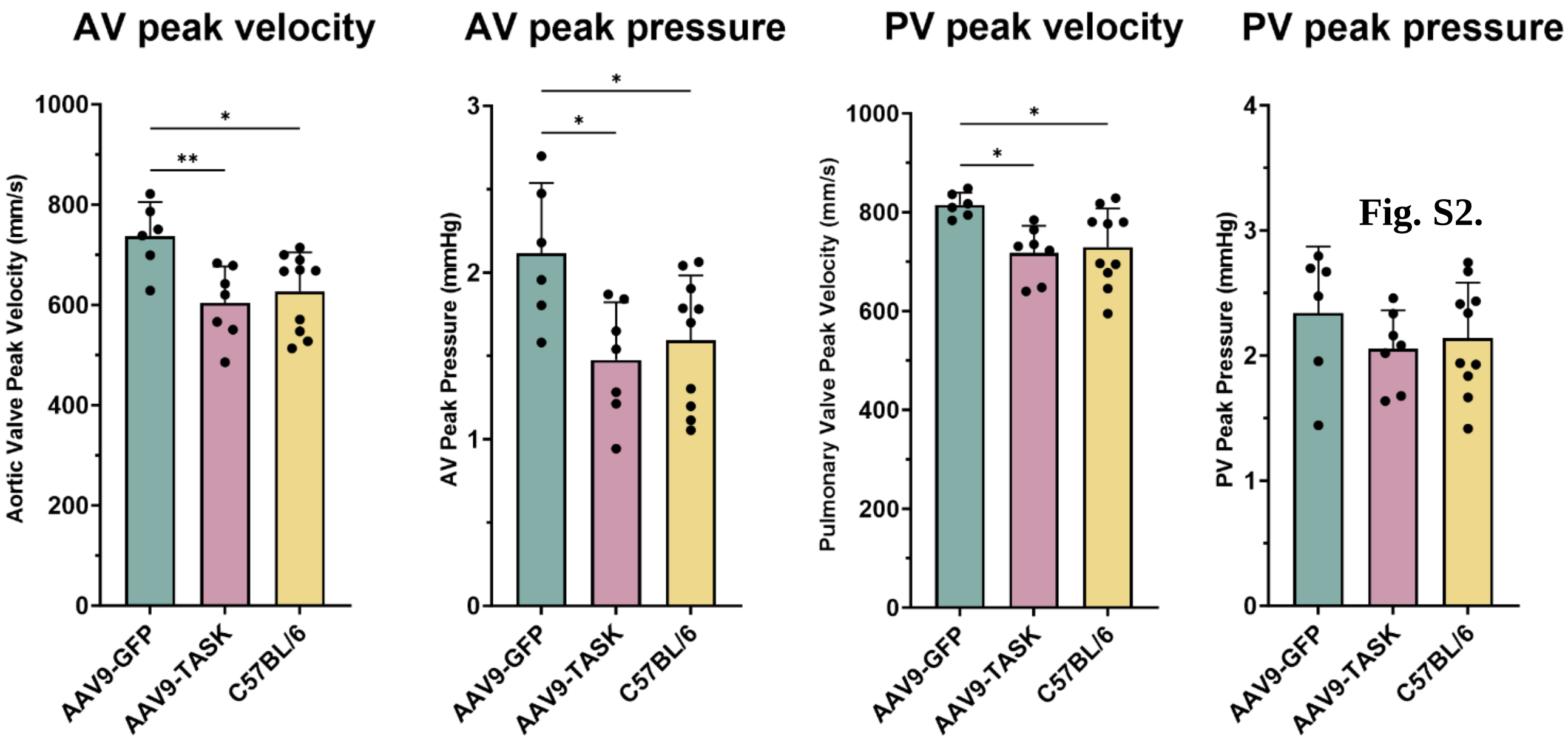

Figure S2: Additional measurements of heart function indicating improvements in AAV9-TASK-treated animals. One-way ANOVA, followed by Dunnett's post hoc multiple Comparisons test (∗∗∗∗$p < 0.0001$, ∗∗∗$p < 0.001$, ∗∗$p < 0.01$ and ∗$p < 0.05$; mean ± SD; n = 6-10 mice).

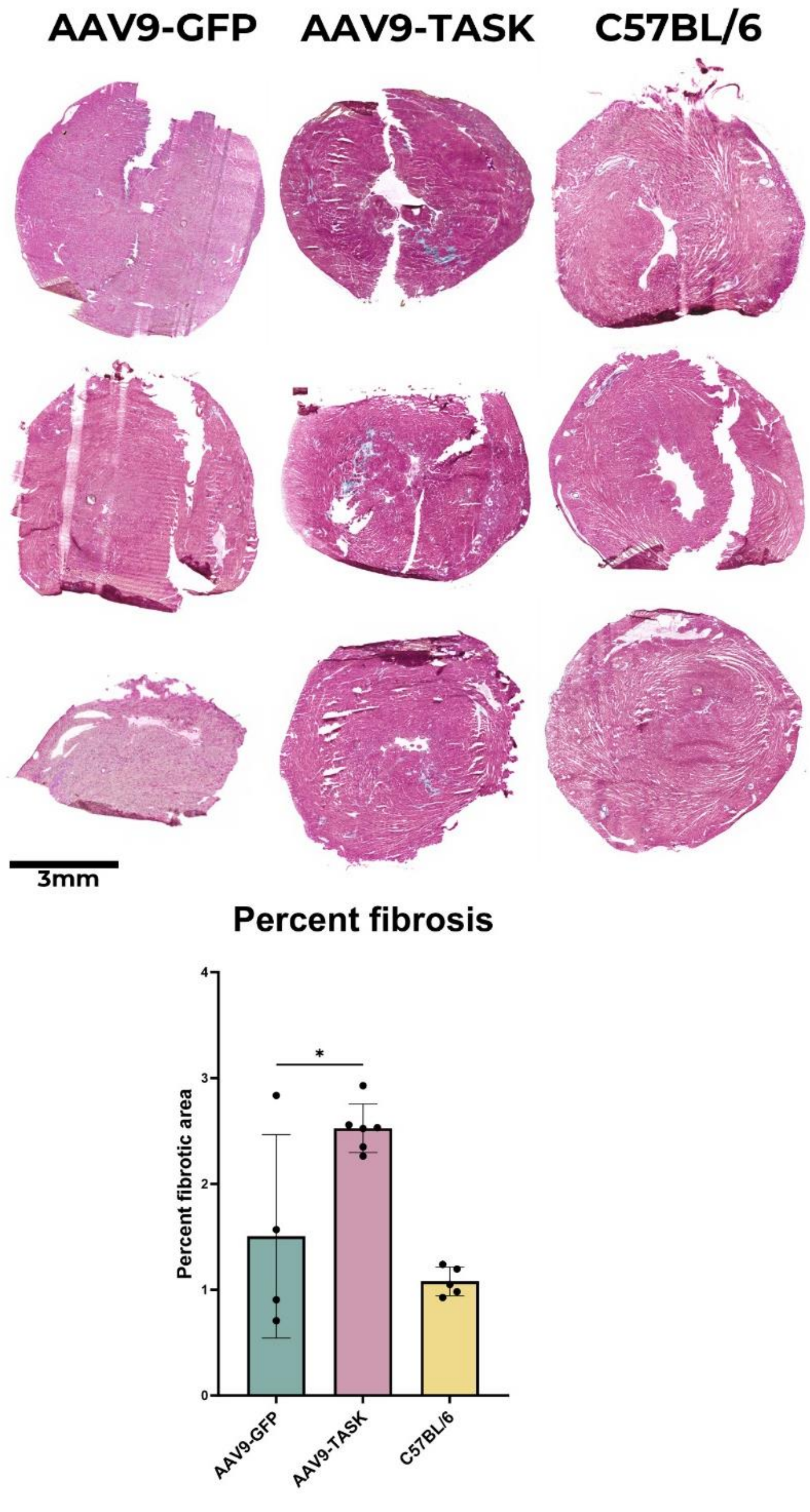

Figure S3: Heart cryosections stained with Masson's trichrome and total fibrotic area quantified (scale bar, 3mm, each dot represents mean of 3-5 images per mouse). One-way ANOVA, followed by Dunnett's post hoc multiple comparisons test (∗∗∗∗p < 0.0001, ∗∗∗p < 0.001, ∗∗p < 0.01 and ∗p < 0.05; mean ± SD; n = 4-6 mice).

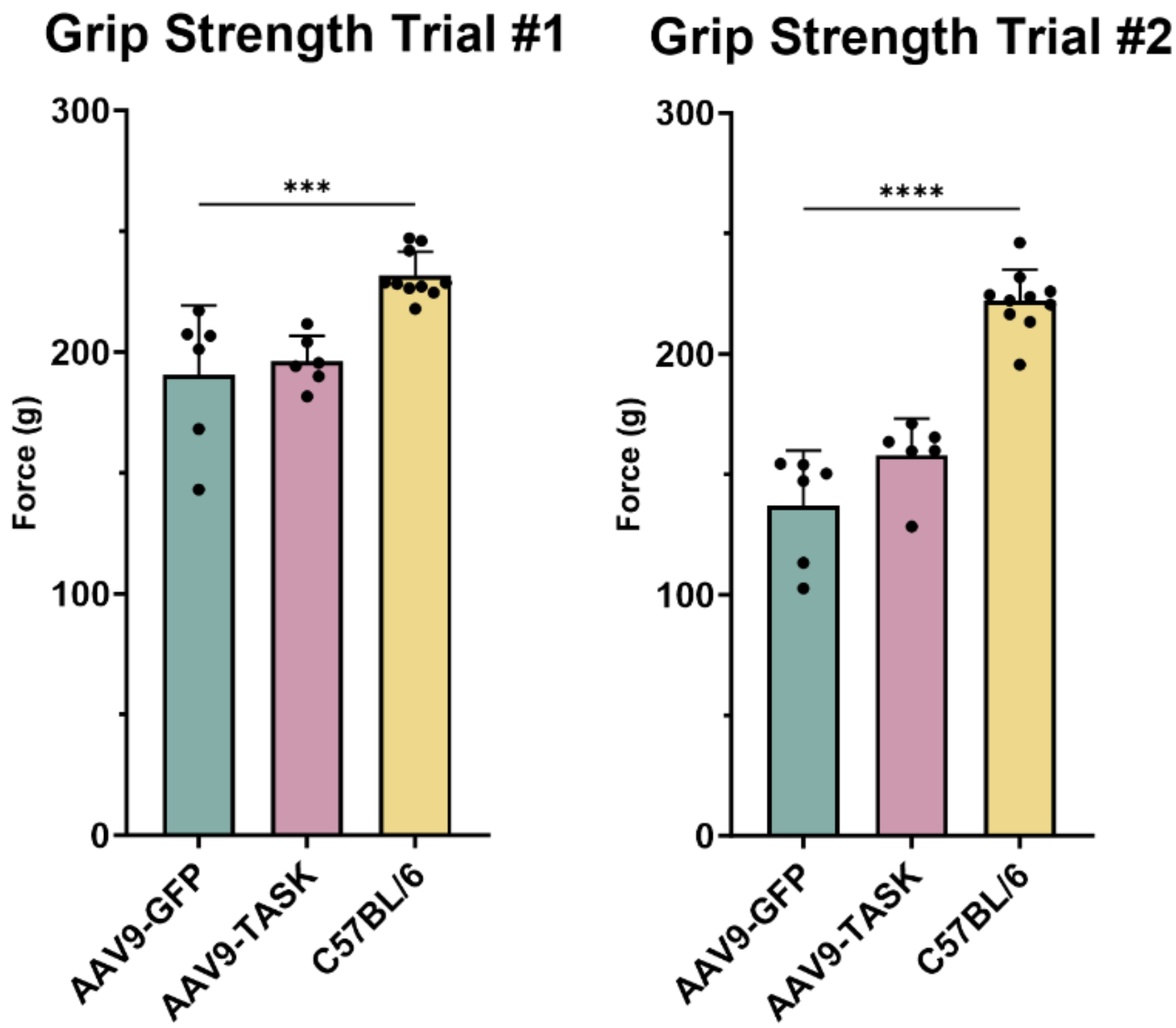

Figure S4: Non-normalized forelimb and hindlimb grip strength test of AAV9-TASK-treated animals. One-way ANOVA, followed by Dunnett's post hoc multiple comparisons test (∗∗∗∗p < 0.0001, ∗∗∗p < 0.001, ∗∗p < 0.01 and ∗p < 0.05; mean ± SD; n = 6-10 mice).

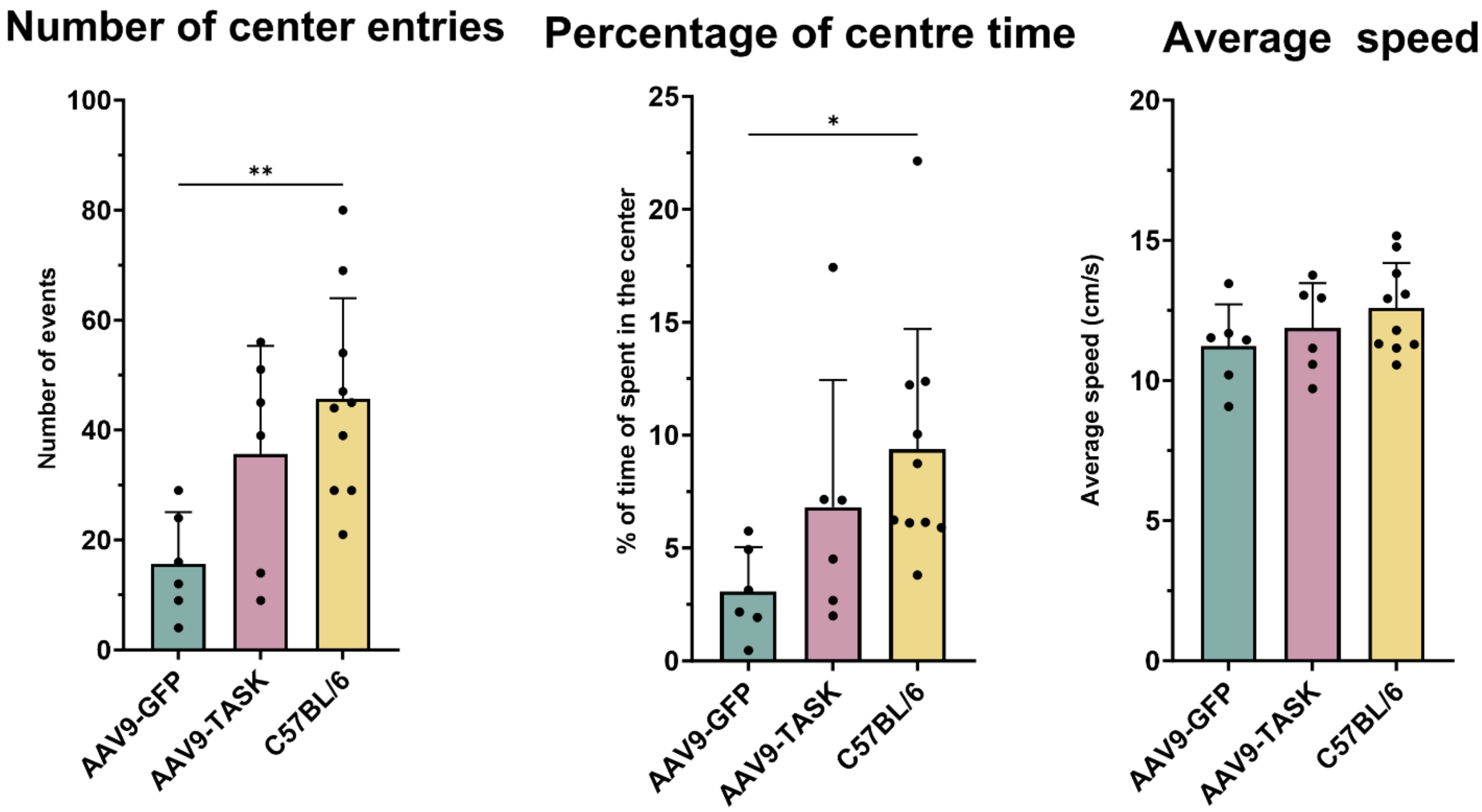

Figure S5: Additional open field test results indicate positive trends in mobility for systemically treated *Dmd Δ52-54* mice. One-way ANOVA, followed by Dunnett's post hoc multiple comparisons test (∗∗∗∗p < 0.0001, ∗∗∗p < 0.001, ∗∗p < 0.01 and ∗p < 0.05; mean ± SD; n = 6-10 mice).

Movie S1.
B mode video of C57BL/6 mouse heart (left ventricle).

Movie S2.
B mode video of AAV9-GFP treated *Dmd Δ52-54* mouse (left ventricle).

Movie S3.
B mode video of AAV9-TASK treated *Dmd Δ52-54* mouse (left ventricle).
